# Reassessing the role and lifetime of Q_*x*_ in the energy transfer dynamics of chlorophyll *a*†

**DOI:** 10.1039/d4sc06441k

**Published:** 2024-11-27

**Authors:** Erika Keil, Ajeet Kumar, Lena Bäuml, Sebastian Reiter, Erling Thyrhaug, Simone Moser, Christopher D. P. Duffy, Regina de Vivie-Riedle, Jürgen Hauer

**Affiliations:** a Technical University of Munich, TUM School of Natural Sciences, Department of Chemistry Lichtenbergstrasse 4 85748 Garching Germany juergen.hauer@tum.de; b Department of Chemistry, Ludwig-Maximilians-Universität München Butenandtstr. 11 81377 Munich Germany; c Institute of Pharmacy, Department of Pharmacognosy, University of Innsbruck Austria; d Digital Environment Research Institute, Queen Mary University of London London E1 4NS UK

## Abstract

Chlorophylls are photoactive molecular building blocks essential to most photosynthetic systems. They have comparatively simple optical spectra defined by states with near-orthogonal transition dipole moments, referred to as B_*x*_ and B_*y*_ in the blue/green spectral region, and Q_*x*_ and Q_*y*_ in the red. Underlying these spectra is a surprisingly complex electronic structure, where strong electronic-vibrational interactions are crucial to the description of state characters. Following photoexcitation, energy-relaxation between these states is extremely fast and connected to only modest changes in spectral shapes. This has pushed conventional theoretical and experimental methods to their limits and left the energy transfer pathway under debate. In this work, we address the electronic structure and photodynamics of chlorophyll *a* using polarization-controlled static – and ultrafast – optical spectroscopies. We support the experimental data analysis with quantum dynamical simulations and effective heat dissipation models. We find clear evidence for B → Q transfer on a timescale of ∼100 fs and identify Q_*x*_ signatures within fluorescence excitation and transient spectra. However, Q_*x*_ is populated only fleetingly, with a lifetime well below our ∼30 fs experimental time resolution. Outside of these timescales, the kinetics are determined by vibrational relaxation and cooling. Despite its ultrashort lifetime, our theoretical analysis suggests that Q_*x*_ plays a crucial role as a bridging state in B → Q energy transfer. In summary, our findings present a unified and consistent picture of chlorophyll relaxation dynamics based on ultrafast and polarization-resolved spectroscopic techniques supported by extensive theoretical models; they clarify the role of Q_*x*_ in the energy deactivation network of chlorophyll *a*.

## Abstract 2

Chlorophylls are essential photosynthetic pigments. We provide a simple and consistent interpretation of their surprisingly complex energy deactivation mechanism, based on tailored spectroscopic methods and state-of-the-art theoretical techniques.

## Graphical abstract

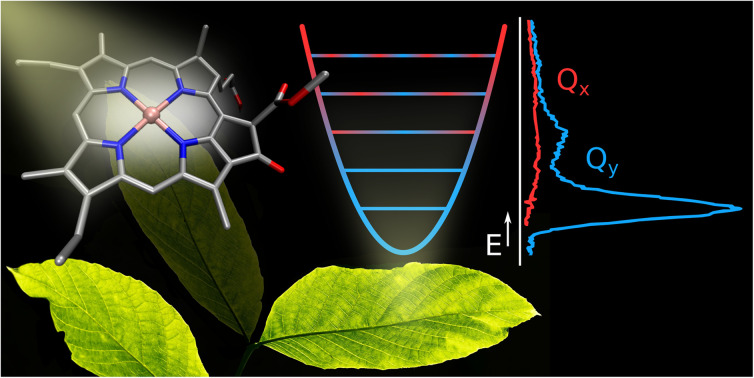

## Introduction

Photosynthetic systems efficiently harness sunlight by absorbing it and transferring the excitation energy to a reaction center where charge separation occurs.^1,2^ Globally, the vast majority of light-harvesting and charge-separation functionality relies on chlorophylls (Chls) or bacteriochlorophylls (BChls). They play essential roles in energy transfer and charge separation and serve as the principal cofactors in the early steps of photosynthesis. For this reason, much effort has been devoted to understanding their electronic structure and ultrafast relaxation dynamics using various experimental and theoretical methods.^3–8^

**Fig. 1:**
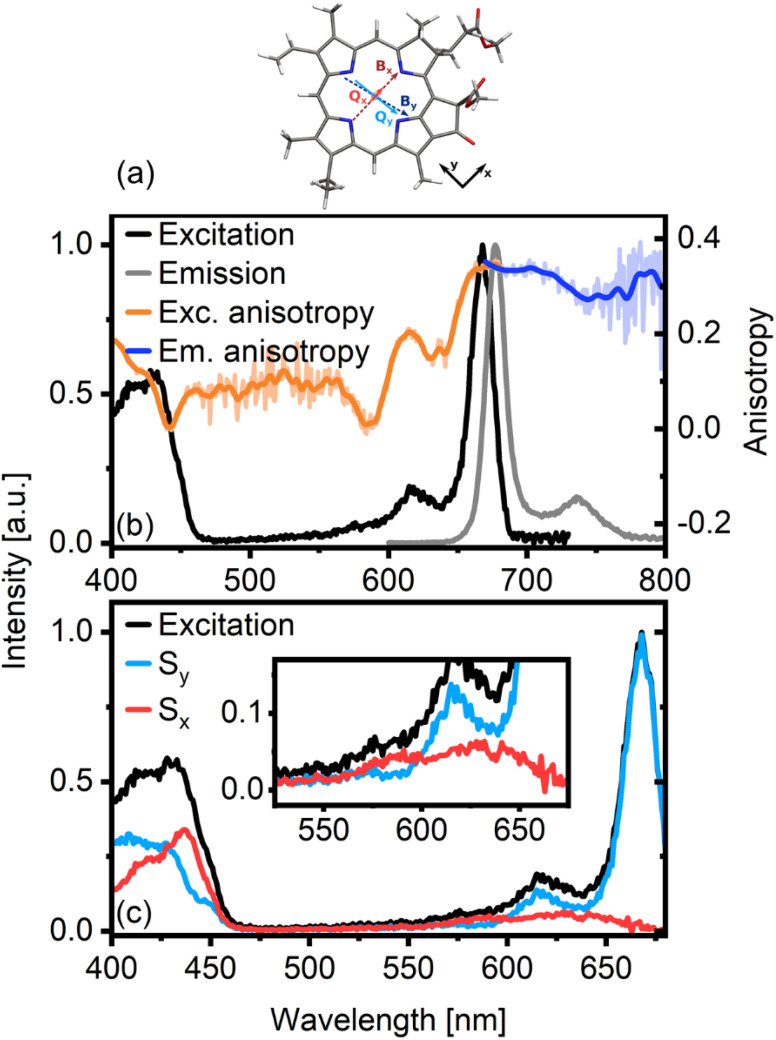
Molecular structure of Chl *a* including the relative orientations of the main transition dipole moments along the porphyrin rings (a). There are two sets of optical transitions (B and Q), each comprising two almost perpendicular TDMs (subscripts *x* and *y*). Panel (b) shows the excitation and emission anisotropy of Chl *a* in an isopropanol glass. The emission anisotropy (*r*_em_, blue line) was measured for *λ*_exc_ = 660 nm, while the excitation anisotropy (*r*_exc_, orange line) was measured for *λ*_em_ = 690 nm. The excitation anisotropy clearly shows the presence of differently oriented transitions. We therefore decompose it into *x*-polarized (S_*x*_) and *y*-polarized (S_*y*_) excitation spectra (c) for *β* = 17°.

Despite their structural diversity, all Chls share two prominent spectral bands: the lowest energy Q-band (550–720 nm) and the Soret- or B-band (350–480 nm). Historically, these bands have been characterized using the Gouterman four-orbital model of the π–π* transitions in porphyrins.^9,10^ In this model, the Q-band consists of two distinct electronic states, which we name Q_*x*,el_ and Q_*y*,el_. Their indices relate to the direction of the respective transition dipole moment within the plane of the macrocycle (Fig. 1a). Later, increasingly sophisticated quantum chemical calculations and experiments aimed to refine the assignment of the observed absorption bands to the two electronic states. However, the energetic position of the Q_*x*,el_ state remained ambiguous. In 2013, Reimers *et al.*^3^ proposed a new band assignment based on vibronic coupling, mixing the states within the Q-band. We refer to the states after incorporating such coupling effects as Q_*x*_ and Q_*y*_. This coupling strongly influences the spectral properties of Chl *a*. For example, the angle between the Q_*x*_ and Q_*y*_ transition dipole moments would naively be expected to be close to 90°, but the experimentally measured value for Chl *a* is ∼70–78°.^11,12^ In several subsequent two-dimensional electronic spectroscopy (2DES) studies, vibronic coupling was discussed as the basis of fast signal oscillations,^13,14^ the vibronic origin of which was proven by polarization-controlled studies.^7^ A similar conclusion was reached from theoretical studies, where it was shown that the Q_*x*_ and Q_*y*_ potential energy surfaces do not cross in an energetically accessible region, and non-adiabatic coupling facilitates ultrafast population transfer across the potential energy surfaces.^5^ Previous to 2DES studies, results from transient absorption (TA) spectroscopy were interpreted so that Q_*x*_ → Q_*y*_ transfer shows a strong and unusual solvent dependence, with Q_*x*_-lifetimes ranging from 100–250 fs in different solvents.^8,15^

In this work, we reinterpret the internal conversion dynamics of Chl *a*, as determined by TA spectroscopy with ∼20 fs excitation and ultra-broadband probing. To offer a comprehensive picture of the relevant deactivation pathways in Chl *a*, we perform TA experiments exciting the B and the Q-band selectively and compare our results with theoretical models. We can unambiguously determine a B → Q transfer time of ∼100 fs from our experimental data and corroborate our findings using theoretical models. We also show that coupling effects within the Q bands lead to almost instantaneous intraband relaxation.

To test our hypothesis of near-instantaneous Q_*x*_ → Q_*y*_ transfer, we go beyond global analysis of TA data and search for Q_*x*_-signals using polarization-selective spectroscopy, followed by isolation of polarization-associated spectra.^16^ Given reported Q_*x*_ lifetimes of up to several hundreds of femtoseconds,^15^ we should observe x-polarized stimulated emission (SE) from Q_*x*_, along with Q_*x*_ ground-state bleach (GSB) and excited-state absorption (ESA). While we successfully isolate the GSB signature of Q_*x*_, we find no sign of *x*-polarized SE or ESA features to indicate a transiently populated Q_*x*_ state. We attribute this to vibronic mixing and rule out the conventional interpretation of Q_*x*_ as a state with up to 300 fs lifetime.^15^ Instead, we offer an interpretation of the kinetic components found in TA based on intra-molecular vibrational redistribution and subsequent vibrational cooling, as described by an effective model.

The paper is structured as follows: to identify GSB signatures of Q_*x*_, we first analyze polarization-associated spectra derived from cryogenic fluorescence excitation spectra (“Assignment of Q_*x*_ and Q_*y*_ transitions”). Direct excitation of Q_*x*_ is a non-ideal starting ground for studying dynamics, as the absorption features of Q_*x*_ and Q_*y*_ overlap strongly. Instead, we excite the B-band and try to isolate transient Q_*x*_ features. We compare these results with TA data after direct Q_*x*_/Q_*y*_ excitation (“Ultrafast relaxation dynamics of Chl a”).

Similarly to the dissection of linear spectra, we attempt to isolate Q_*x*_- and Q_*y*_-related excited state absorption features in transient absorption anisotropy (TAA) (“Isolating Q_*x*_-features by polarization control”) and compare our measured results to theoretical predictions (“System and system-bath relaxation dynamics at different timescales”). We show that neither TA experiments with sub 20 fs pump pulses nor polarization-controlled experiments can establish a timescale for Q_*x*_ → Q_*y*_ transfer. Instead, our work offers a new perspective on the energy transfer dynamics in Chl *a*. We relate the experimentally observed ultrafast dynamics to solvent relaxation processes and obtain a robust and consistent picture supporting strong mixing between Q-states.

## Results

### Assignment of Q_*x*_ and Q_*y*_ transitions

Fig. 1b shows the normalized excitation (black line) and emission (grey line) spectra of Chl *a* in an isopropanol glass. The small Stokes shift of the fluorescence spectrum indicates a minor displacement of the excited state potential surface, as also discussed in earlier work.^14,17^ Accordingly, the emission and absorption spectra are similar in shape, although not exactly symmetric. The differences are mainly in the sidebands, where the breakdown in mirror-image symmetry has been connected to vibronic coupling interactions.^17,18^ The excitation spectrum in the Q band region has one dominant peak at *ca.* 670 nm and two shoulders at *ca.* 620 nm and 570 nm. Based on previous work, the main peak can be assigned to the Q_*y*_ transition.^19^ However, assigning the shoulders – particularly determining the position of the Q_*x*_ transition – is not straightforward.^6,17^ The locations of the Q_*x*_ and Q_*y*_ transitions in Chl *a* vary with the solvent and the coordination of the molecule. In isopropanol, where the system is penta-coordinated,^20^ the Q_*x*_ band overlaps with Q_*y*_ vibrational bands,^17^ making a definite assignment of the shoulder bands challenging. Several groups have investigated the transition energy of Q_*x*_ and its vibronic sidebands, either by calculation^4,5^ or experiment.^3,17^ In this work, we employ polarized excitation and emission spectroscopy of Chl *a* in an isopropanol glass.

Alongside the excitation and emission spectra, Fig. 1b also shows fluorescence anisotropy traces. The emission anisotropy (*r*_em_, blue line) was measured for *λ*_exc_ = 660 nm, while the fluorescence excitation anisotropy (*r*_exc_, orange line) was measured for *λ*_Em_ = 690 nm. In the Q band region, r_exc_ of the main transition centered at 670 nm is almost flat. It reaches a value of 0.35, indicating near-parallel emission and absorption dipoles. As emission occurs from Q_*y*_, this band must be *y*-polarized. The same high value is obtained from *r*_em_ upon excitation at 660 nm, proving the measurements to be consistent. While the main transition at 670 nm belongs to Q_*y*_, r_exc_ shows clearly that the high-energy side of the Q band region contains contributions from electronic states with different polarizations, as anisotropy values here are as low as zero.

Several approaches have been developed to decompose optical spectra by leveraging the information on transition dipole moment (TDM) directions available from anisotropies.^21–23^ Most commonly, in the absence of a macroscopically oriented sample, these methods “project” the isotropic spectrum into contributions either parallel or orthogonal to a reference TDM. For excitation anisotropy measurements, this reference is the TDM of the emissive transition, while for emission anisotropy, the reference TDM is that of the pumped transition. In many cases, however, the TDMs of a given molecule are not exactly orthogonal, and the decomposition of the spectra in orthogonal components is not ideal. As earlier work has shown that the Chl *a* Q_*x*_ and Q_*y*_ TDMs are not orthogonal,^11^ this type of spectral decomposition will not result in optimal separation of the contributions. In a recent study, we developed a slightly modified decomposition approach, which is suitable for systems where TDMs are not orthogonal.^16^ In this approach, we can cleanly separate spectral contributions not only when the underlying TDMs are at an angle *θ* = 90°, but at any angle *θ* = 90° − *β*. The parameter angle *β* introduced here can be thought of as the rotation angle of an orthogonal molecular coordinate system defined by the reference TDM. For details, we refer to the corresponding publications.^16,24^

In Fig. 1c we show the decomposition of the Chl *a* excitation spectrum into components polarized parallel (here: S_*y*_, blue in Fig. 1c) and orthogonal (here: S_*x*_, red) to the emissive transition (Q_*y*_ fluorescence) according to the procedure outlined above. We note that besides Q_*x*_ features, S_*x*_ also contains signals with a TDM parallel to B_*x*_, while S_*y*_ contains both Q_*y*_ and B_*y*_ contributions. If we assume orthogonal Q_*x*_ and Q_*y*_ transitions (corresponding to *β* = 0), we clearly achieve sub-optimal separation of the spectrum with noticeable contamination of the S_*x*_ spectrum by features associated with the *y*-polarized transitions (see Fig. S1†). Increasing the value of *β* to 17°, however, maximizes the 670 nm peak in the S_*y*_ spectrum and simultaneously achieves optimal separation of the spectrum into “pure” but overlapping Q_*x*_ and Q_*y*_ contributions, each with their respective vibronic progressions. Note that both the S_*x*_ spectral maximum at approximately 640 nm and the relative angle between Q_*x*_ and Q_*y*_ of *θ* = 90 − *β* = 73° are in good agreement with previously reported results.^4^ In essence, polarization-associated analysis of Chl *a* excitation spectra is a straightforward and effective way of isolating overlapping Q_*x*_ and Q_*y*_ ground state transitions.

### Ultrafast relaxation dynamics of Chl *a*

Although Chl *a* has been studied extensively, open questions remain concerning the exact mechanism of energy transfer dynamics within this molecule. The most common scheme employed to explain Chl *a* energy relaxation is a sequential one, *i.e.*, B_*x*_ → Q_*x*_ → Q_*y*_. B → Q transfer seems to occur within 100–150 fs, as inferred by time-resolved fluorescence depletion spectroscopy^15^ or UV pump, NIR probe TA.^25^ A time-constant in the 100–250 fs range with notable strong and unusual solvent dependence has been attributed to the subsequent Q_*x*_ → Q_*y*_ transfer.^15^

**Fig. 2:**
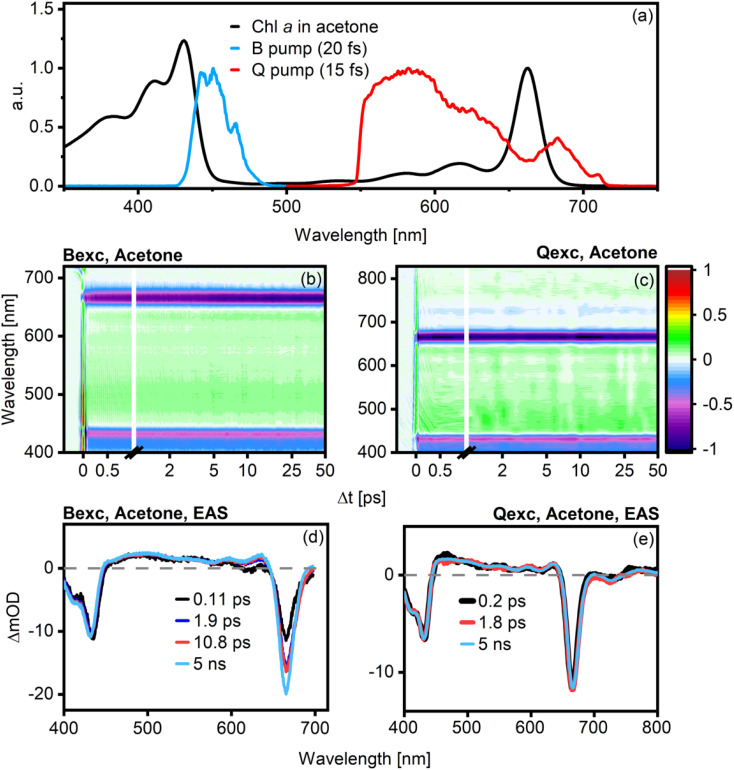
Absorption spectrum of Chl *a* in acetone plotted against the pump spectra for B-band (blue line) and Q-band (red line) excitation (a). Chirp-corrected TA data for both excitation conditions are shown in (b) and (c). Visible changes in the dynamics are present after B excitation, with a clear increase of the Q-band GSB signal over time, but this is not true after Q excitation. The EAS and lifetimes extracted from global analysis of Chl *a* in acetone are shown in (d) after excitation in the B-band and in (e) after excitation in the Q band. The corresponding plots for Chl *a* in EtOH and benzonitrile are shown in Fig. S2.†

To better elucidate the mechanism of energy transfer dynamics in Chl *a*, we perform TA experiments under different excitation conditions (B- and Q-band excitation) and in various solvents (acetone, ethanol (EtOH), and benzonitrile (BN)). In particular, we expect to observe direct evidence of B → Q transfer and to understand the origin of the reported solvent dependence of the Q_*x*_ → Q_*y*_ energy transfer step. In the following, we show a representative set of data for Chl *a* in acetone. The corresponding plots for the other two solvents are shown in ESI Fig S2 and S3.† Absorption and pump pulse spectra are shown in Fig. 2a (Fig. S2†). Chirp-corrected TA data in magic angle (MA) configuration after B- and Q-band are shown in Fig. 2b and c, and the global analysis results are shown in Fig. 2d and e (Fig. S3†). The lifetimes corresponding to the individual spectral components are reported in each panel. Only the evolution-associated spectra (EAS) are shown for simplicity, while the decay-associated spectra for all solvents are shown in ESI Fig. S4.†

In the case of B-band excitation (Fig. 2b and d), the best fit is achieved with four kinetic components. The shortest component has a lifetime of ∼110 fs (black line), with negligible solvent dependence. It is characterized by a decrease and blue shift of the signal in the B-band region (430 nm) and a concomitant increase of the main Q-band signal (660 nm). This behavior is consistent with B → Q transfer, and the B → Q transfer time agrees with previously reported values.^8^ The second component (dark blue line) shows minimal spectral evolution; the only noticeable feature is a slight broadening of the Q_*y*_ band. From the global fits, it appears to be strongly solvent-dependent, varying from *ca.* 400 fs (BN, *cf.* Fig. S3†) to 2 ps (acetone). Notably, the amplitude of this component is near-negligible, and it is not associated with any spectral evolution typical of state-to-state energy transfer. As changes to small-amplitude components do not substantially affect the goodness-of-fit, the lifetime associated with this component is inevitably highly uncertain. As such, although this component appears to exhibit some solvent dependence, we do not make strong quantitative claims about this behavior. We emphasize, however, that this component does not contain significant changes to stimulated emission or excited state absorption, implying that it is not related to population transfer. The third kinetic component (red line), with a lifetime of about 7–11 ps, correlates with a narrowing of the Q_*y*_ band and an increase in its amplitude. Finally, the last kinetic component (light blue line, fixed to 5 ns) is readily assigned to the excited state lifetime of Chl *a* as known from fluorescence lifetime measurements.^26^

In the case of Q-band excitation (Fig. 2c and e), only three components are necessary to obtain a satisfactory data fit. We again observe an ultrafast component of 100–300 fs (black line) that was previously assigned to Q_*x*_ → Q_*y*_ transfer.^8,15^ If this component was associated with population transfer, we would expect a signal decrease in the 520–630 nm Q_*x*_-region concomitant with an increase in the Q_*y*_ ground-state bleach (GSB)/stimulated emission (SE) amplitude around 660 nm. As in the B-band excitation case, however, we do not observe any such features, leaving this component inconsistent with a population transfer process. An intermediate 1.5–15 ps component (red line) with extremely small amplitude similarly does not relate to significant spectral changes, suggesting a small structural reorganization process. The longest component is again the excited state lifetime of Chl *a*, fixed at 5 ns (light blue line).

### Isolating Q_*x*_ features by polarization control

General problems with the analysis of the Q_*x*_ features of Chl *a* are the weakness of the transition and its strong spectral overlap with the Q_*y*_-related signals (*cf.*Fig. 1). It could then be conceivable that the lack of Q_*x*_ → Q_*y*_ population transfer features in MA-TA stems from their smaller amplitudes being covered by the larger Q_*y*_ signals.

**Fig. 3:**
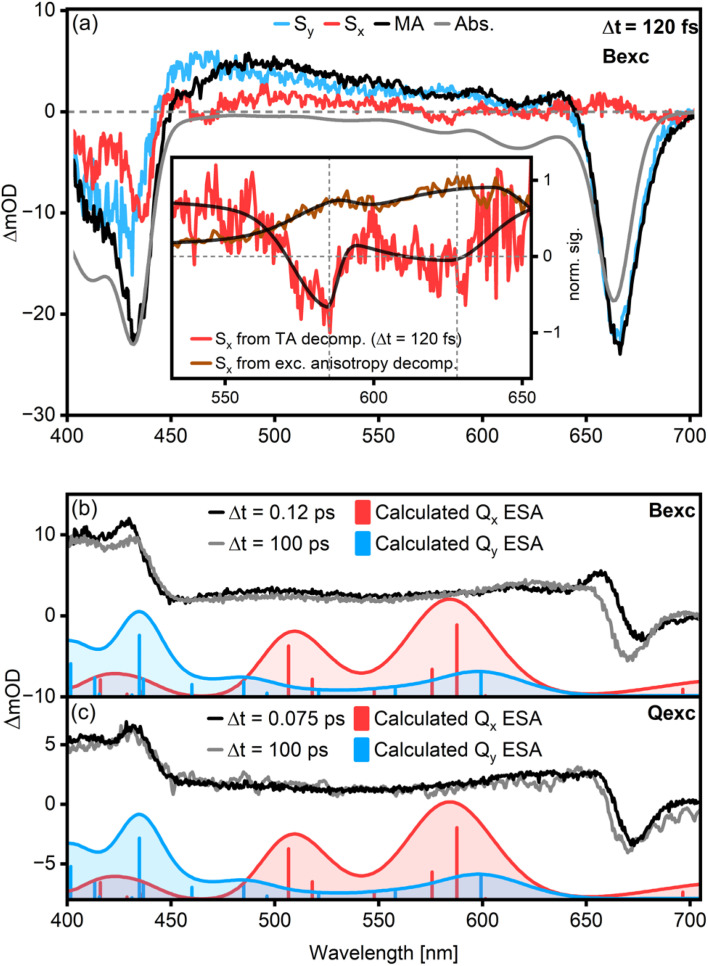
Results of the Chl *a* TA data decomposition into polarized components at Δ*t* = 120 fs (a) for *β* = 32°. Here, S_*x*_ is chosen to indicate the direction parallel to the pumped TDM and contains all contributions with this polarization. Accordingly, the Q_*y*_ transitions are suppressed and appear only in S_*y*_. The Q_*x*_ GSB transitions extracted from polarized TA and steady-state anisotropy match well (a, inset). They show an energy spacing of about 1100 cm^−1^ as calculated by fitting a bi-gaussian function to the peaks. Excited-state associated spectra of Chl *a* after excitation in the B-band (b) and the Q-band (c) were calculated for different time delays (grey and black lines) and are compared to theoretical Q_*x*_ and Q_*y*_ ESA band positions (blue and red histograms and curves, oscillator strength unscaled). The theoretical ESA band positions were calculated considering one acetone molecule coordinating to the central Mg of a Chl *a* molecule. Calculated ESA transitions from Q_*y*_ match the experimental spectrum well, while Q_*x*_-ESA features are absent.

To isolate Q_*x*_-signatures and to determine their transient behavior, we have measured transient absorption anisotropy (TAA) data for Chl *a* in acetone after B-band excitation. With these data, we can decompose the TA signals into polarized components in an analogous procedure to that described for excitation anisotropy.^16,24^ In this case, a value of *β* = 32° is necessary to fully suppress the Q_*y*_ peak. In the employed representation, S_*x*_ contains signals with a TDM parallel to that of B_*x*_, including all Q_*x*_ features, while S_*y*_ contains the Q_*y*_ and B_*y*_ contributions. Representative spectra are shown for a time delay Δ*t* = 120 fs in Fig. 3a. At this delay time, effects due to pump-and-probe pulse overlap are negligible, and there is already population in the Q-band resulting from B → Q transfer (*cf.*Fig. 2b and d).

In search for Q_*x*_-features, we identify two weak negative peaks at 580 nm (*ca.* 17 200 cm^−1^) and 625 nm (*ca.* 16 000 cm^−1^), matching the Q_*x*_ features observed in the excitation anisotropy measurements (Fig. 3a, red line and inset). They show an energy spacing of about 1100 cm^−1^ as calculated by fitting a bi-gaussian function to the peaks (Fig. 3a, inset, black lines), in excellent agreement with magnetic circular dichroism spectra for pentacoordinated Chl *a*.^3^

The lineshape of the S_*x*_ spectrum remains identical for time delays between 100 fs and several ps (Fig. S5†). This means that while we observe the Q_*x*_ GSB, no Q_*x*_ SE (or excited-state absorption (ESA)) features are discernible. We support this statement further by performing a global analysis of the polarization-decomposed TA maps shown in the ESI (Fig. S6†). Similar time constants are needed to fit S_*x*_ and S_*y*_, and there is no evidence for a transient population of an excited Q_*x*_ state.

We further examine the possibility of Q_*x*_ ESA features by comparing the experimental excited state spectrum to calculations for both B-excitation (Fig. 3b) and Q-excitation (Fig. 3c). The experimental excited state spectrum is determined from the measured TA spectrum by subtracting the GSB.^27,28^ The latter is approximated by a scaled absorption spectrum. The resulting spectra only contain ESA and SE contributions. We then compare the lineshapes of these spectra at early and late times with calculations for the Q_*x*_- and Q_*y*_-associated ESA, considering one acetone molecule coordinating to the central Mg of a Chl *a* molecule. Notably, the excited-state spectrum at early times after B-band excitation has a positive feature at 655 nm which is absent at later times and in the case of Q-band excitation. We attribute this to ESA from the B-band, which is cancelled by the Q-band GSB, explaining why we do not observe this feature in the magic angle TA data in Fig. 2d. While the calculated Q_*y*_ ESA-peaks correspond to measured features at both early and late times, the features related to Q_*x*_ ESA are missing. In particular, two intense Q_*x*_-features are expected in the 16 000–21 000 cm^−1^ region at early times. However, the measured spectra in this region at early and late times are effectively identical. While ESA from Q_*y*_ is clearly visible, the lack of excited-state signatures associated with Q_*x*_ means that Q_*x*_ is either not significantly involved in the energy relaxation network or its lifetime is shorter than the experimental time resolution.

### System and system-bath relaxation dynamics at different timescales

**Fig. 4:**
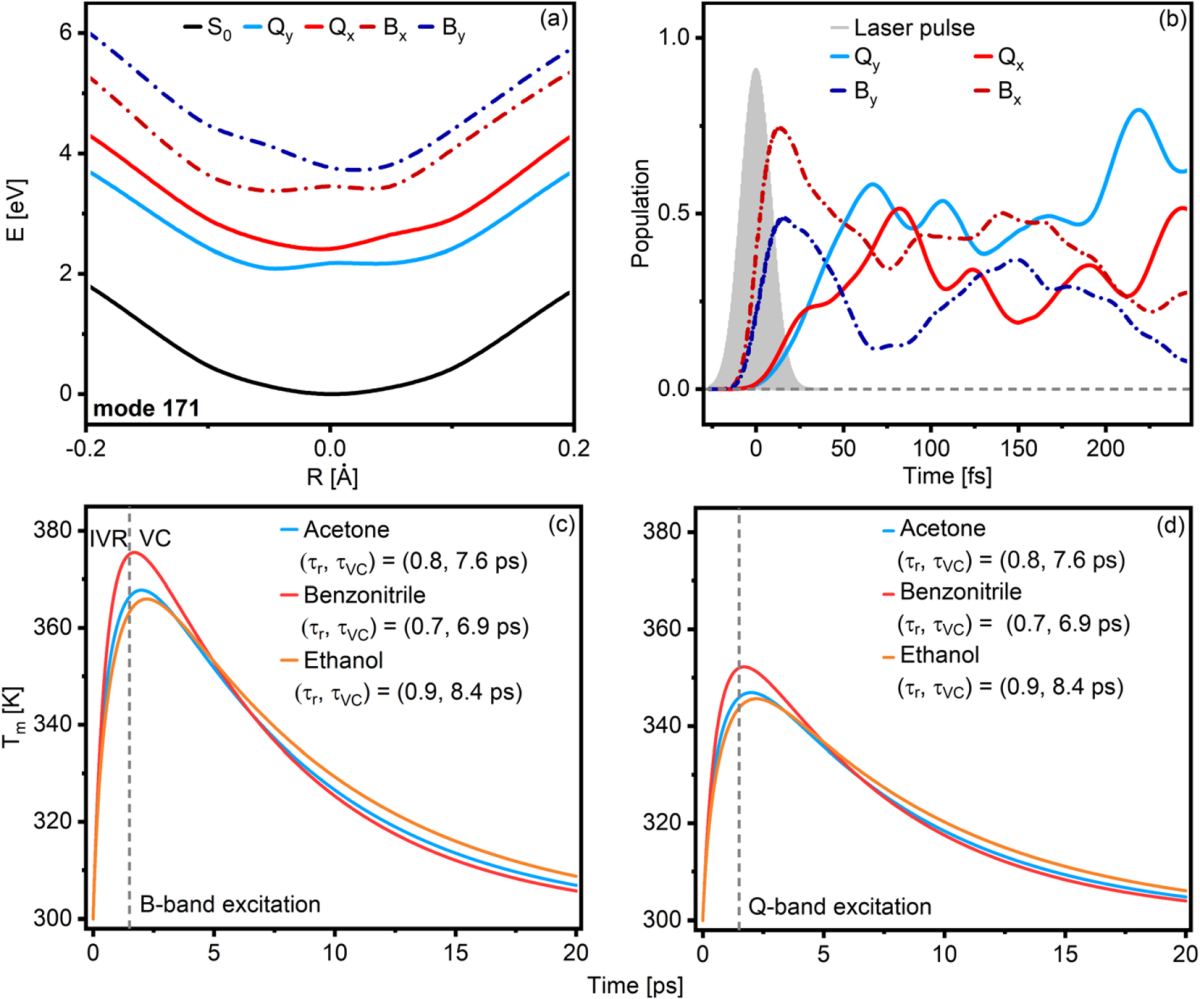
Cuts through the potential energy surfaces of Chl *a* along the mode with the strongest projection on the non-adiabatic coupling vector (a). The curve displacement between the excited states is negligible, resulting in nested potentials with no evident curve crossings. B_*x*_ and B_*y*_, as well as Q_*x*_ and Q_*y*_, show a small energy gap and therefore large coupling, which explains the almost instantaneous population transfer between them. The comparatively large energy gap between B_*x*_ and Q_*x*_, on the other hand, leads to predicted transfer times of ∼100 fs (b). At longer timescales, energy relaxation occurs *via* intra-molecular vibrational redistribution (IVR), followed by slower equilibration with the surrounding solvent (vibrational cooling VC). We capture the effects of biphasic energy relaxation in a simplified model for B-band (c) and Q-band (d) excitation scenarios and for different solvents. Since less energy is deposited into the Q_*y*_ vibrational manifold, the peak local temperature is less than for B-band excitation.

We compare our experimental results with quantum dynamics calculations (details in Fig. S7, S8 and Table T1†). Our XMS-CASPT2 results predict that the excited-state potentials of Chl *a* are nested on top of each other with minimal curve displacement. This is illustrated in Fig. 4a with a cut through the potential energy surface (PES) along the mode with the highest projection onto the non-adiabatic coupling (NAC) vector, with a vibrational frequency of 1489 cm^−1^ (mode 171 in the frequency analysis). Cuts along modes with weaker coupling and different frequencies show the same trend (Fig. S8†). We did not observe any crossings in the central region of the PES where most of the population resides. The temporal evolution of the population of the adiabatic states is depicted in Fig. 4b. We note that while the dynamics simulations include the non-adiabatic coupling terms, the depicted populations correspond to those of the electronic states within the Born–Oppenheimer approximation and not the vibronic states observed in experiment. After excitation to the B-band with an explicitly simulated pump pulse, a population transfer from B to Q occurs on a moderately longer timescale than the intra-band transfer. This is explained by the significant energy difference between the B and Q bands (Fig. 4a). The exact timescale of the B → Q transfer depends on the amount of initially excited vibrational levels contained in the wavepacket. On PES constructed with normal modes of lower overlap with the NAC vector and in different spectral ranges, the dynamics are generally slower than normal modes of high overlap (Fig. S8†). Nonetheless, all simulations have in common that the intra-band transfer in both the B and the Q band is almost instantaneous. In contrast, the B to Q transfer takes about 50–150 fs. This is in excellent agreement with our experimental data presented in the discussion of Fig. 2. Further, we find that the B → Q transfer heavily depends on coupling the B band with Q_*x*_. If this coupling is turned off, the B → Q transfer slows down significantly. On the contrary, if B/Q_*y*_ coupling is turned off, the population dynamics do not change much (Fig. S8†).

While we can readily explain ultrafast time constants with our quantum dynamics simulations, the dynamics at longer times are not practically accessible with this type of calculation. At these longer timescales, we expect energy redistribution and dissipation to the solvent to be the dominating contributions to the dynamics.^28^ As such, the bath must be considered when trying to reproduce the longer kinetic components observed in TA.

We construct a simplified numerical model to test our hypothesis of cooling dynamics on multiple timescales. Excitation of Chl *a* with a visible laser pulse always means that a large amount of energy is deposited into the vibrational modes of the molecule. Energy deposited – directly or indirectly – into a high-lying state of Q_*y*_ will relax in a non-linear sequence of intra-molecular vibrational redistribution (IVR) events, solute-to-solvent energy transfer, and solvent equilibration. Using an effective molecular temperature *T*_m_ (details and modeling parameters in the ESI text, Table T2, Fig. S9 and S10†) that increases with the energy deposited into the system, we predict a molecule-to-solvation shell transfer time of ∼1 ps. This is consistent with observations from our previous work on carotenoids.^29–31^ The dynamics of *T*_m_ are determined by two timescales: *τ*_IVR_ ∼ 1 ps, which accounts for the rise in *T*_m_ due to thermalization of the Q_*y*_ state, and *τ*_VC_ ∼ 7–9 ps, which is the relaxation of the solute due to vibrational cooling (VC) into the solvation shell. The exact values of the time constants vary with the solvent (Fig. 4c and d). The results indicate that the relaxation kinetics are almost independent of whether one excites Q_*y*_ directly or indirectly *via* the B-band. However, the maximum *T*_m_ reached should be affected by excitation wavelength, as this defines the amount of energy that thermalizes (Fig. 4c and d). As *τ*_IVR_ is associated with heating and *τ*_VC_ with cooling, we expect to observe thermal broadening effects of the Q_*y*_-band over ∼1 ps as the maximum *T*_m_ is achieved, followed by spectral narrowing over ∼10 ps as the population of low-frequency vibrational modes drops while the solute and first solvation shell re-equilibrate with the bulk solvent. Careful inspection of the decay-associated and evolution-associated spectra (DAS and EAS, respectively) in the Q-band region (Fig. S11†) confirms these predictions for excitation in the B-band, although the observed lineshape changes are minimal. After Q-band excitation, IVR and cooling are much less pronounced. In particular, we do not observe a narrowing in ∼10 ps as expected. Instead, we observe a minimal but continuous redshift typical of Stokes shift dynamics (Fig. S11†).^28^

## Discussion

The results presented in this work clarify some essential aspects of the mechanism of energy relaxation in Chl *a*. When excited in the B-band, population transfer to the Q band occurs within 120 fs, as demonstrated by TA experiments (Fig. 2d) and quantum dynamical calculations (Fig. 4b). The Q band contains Q_*x*_ and Q_*y*_, two distinct but strongly coupled states with overlapping but discernible absorption spectra (Fig. 1). However, polarized TA measurements show no evidence of *x*-polarized excited state features (Fig. 3). As such, we can conclude that either Q_*x*_ is not involved in B → Q energy relaxation in Chl *a*, or that the Q_*x*_ → Q_*y*_ transfer is faster than our time resolution (*ca.* 30 fs). Our quantum chemical calculations strongly support the latter interpretation, as the B → Q transfer was shown to slow significantly if the B/Q_*x*_ coupling is turned off (Fig. S8†).

An implication of these observations is that none of the time constants found in MA-TA (*cf.*Fig. 2) can be assigned to Q_*x*_ → Q_*y*_ transfer. The small amplitudes and only subtle influence on the spectra of the kinetic components other than the one related to B → Q transfer suggest instead an association with IVR and subsequent heat dissipation to the solvent.

**Fig. 5:**
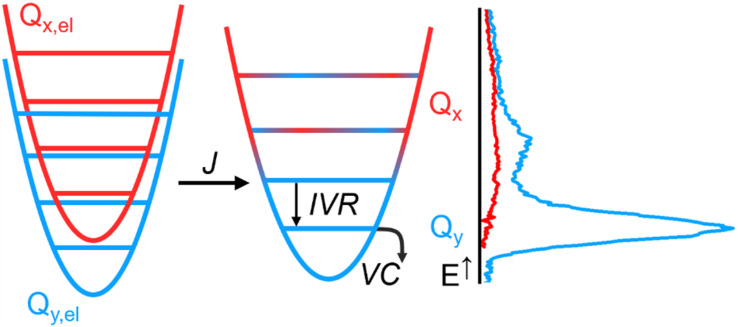
Vibronic mixing (*J*) in the Q band leads to strongly coupled states Q_*x*_ (red) and Q_*y*_ (blue). Ultrafast transfer *via* Q_*x*_ populates a vibrationally excited Q_*y*_, which in turn relaxes *via* intra-molecular vibrational redistribution (IVR), followed by slower equilibration with the surrounding solvent (vibrational cooling VC). The right panel shows a sketch of the spectral profiles of Q_*x*_ and Q_*y*_, as discussed in Fig. 1.

In Fig. 5, we propose a comprehensive scheme for the internal conversion dynamics of Chl *a*. After the initial B → Q transfer, the state we detect is y-polarized. Therefore, if Q_*x*_ is participating in the energy transfer as suggested by the quantum dynamics calculations, its relaxation towards a vibrationally hot, *y*-polarized state must be faster than our time resolution. The relaxation from there to the vibrationally relaxed Q_*y*_ occurs through IVR, leading to heat exchange with the first solvation shell. Further relaxation to bulk solvent (VC) then occurs on slower timescales. Overall, IVR and VC are sufficient to explain the timescales in the evolution of the Chl *a* TA spectra beyond the first few hundred fs after B-band excitation: ∼1 ps for IVR to a hot pseudo-thermal state, ∼10 ps for relaxation of the hot thermal state (Fig. 4c and *cf.*Fig. 2d). The effect of IVR and VC on the lineshape is much less pronounced after Q-band excitation. In this case, the changes in the lineshape can be explained by Stokes shift dynamics.^28^

In summary, our findings shed new light on the internal conversion dynamics in Chl *a*. Although the heating–cooling cycle on the Q_*y*_ surface of Chl *a* is highly complicated and requires further investigation from both experimental and theoretical sides, we reach a good agreement between model and experiment. We present experimental and theoretical evidence for B → Q transfer on a ∼120 fs timescale. The relaxation dynamics in the Q band are dominated by the strong coupling of the Q_*x*_ and Q_*y*_ states and occur on a <30 fs timescale. This leads to the absence of *x*-polarized excited-state signatures in TAA experiments. We attribute this ultrafast Q_*x*_ lifetime to the small and internal-coordinate-independent energy gap between Q_*x*_ and Q_*y*_. Regardless of its short lifetime, Q_*x*_ seems important for efficient B → Q relaxation, as the B → Q transfer rate slows down if B/Q_*x*_ coupling is deactivated. Based on these results, we reinterpret the solvent-dependent, longer time constants found in TA measurements as a combination of IVR, VC, and possibly Stokes shift dynamics on Q_*y*_. Overall, our unique combination of extensive theoretical modeling, numerical methods, and highly sensitive TA experiments allows us to paint a clear and consistent picture of the energy transfer in Chl *a* under the excitation conditions relevant to photosynthetic systems and to shed light on the previously controversial role of the Q_*x*_ state in the Chl *a* dynamics.

## Materials and methods

### Materials

HPLC and spectroscopy grade solvents acetonitrile (ACN), ethyl acetate (EtOAc), acetone, ethanol (EtOH), BN and methanol (MeOH) were obtained from VWR (Ismaning, Germany) and ultra-pure water (18 MΩ cm^−1^) from a Millipore S.A.S. Milli-Q Academic system (18.2 MΩ cm^−1^, Molsheim, France).

### Chromatography

#### Analytical HPLC

An Agilent 1260 Infinity II LC system was used with a 1260 Infinity Degasser, a 1260 Series quaternary pump and 1260 Series diode array detector; Merck LiChrospher® 100 RP-18 (5 μm) LiChroCART® 125-4, with or without a Phenomenex ODS 4 × 2 mm i.d. pre-column; injection volume: 100 μL. Isocratic mobile phase: ACN/MeOH/EtOAc 6/2/2,^32^ flow rate 0.5 mL min^−1^. Data were processed with OpenLab CDS Data Analysis 2.3.

#### Semi-preparative HPLC

Büchi Pure C-830 with prep HPLC pump 300 bar, fraction collector, and prep sample injection valve. Gynkotek LC-System with manual sampler, M480 pump, Phenomenex DG-301 online degasser, Gynkotek UVD 640 diode array detector and a Rheodyne injection valve with 5 mL loop. Column: Phenomenex Luna C8(2), 100 Å, 250 × 100 mm with a Phenomenex pre-column C18 15 × 21.2 mm; mobile phase ACN/MeOH/EtOAc 6/2/2, isocratic conditions, flow rate 4 mL min^−1^. Data were processed with Gynkosoft 5.50 or Büchi Pure software 1.5.

### Spectroscopy

HR-MS were measured at the MS facility of the Department of Chemistry, University of Munich. Data were processed using Xcalibur.

UV-vis spectra were measured on a PerkinElmer Lambda 365 spectrometer. Emission spectra were measured on a Spectrofluorometer FS5 (Edinburgh Instruments). All spectra are background-corrected.

### Extraction procedure for Chl *a*

Frozen spinach was purchased at a local supermarket (Edeka junger Spinat). 8 portions of frozen spinach (approximately 15 g each) were thawed by soaking the spinach in water; water was removed from the soaked spinach by squeezing the spinach with a table cloth. The spinach was then mixed with a blender in 100 mL of cold acetone/MeOH (9/1 v/v). The resulting slurry was centrifuged at 4 °C for 5 min at 1000 rpm. The supernatant was filtered; the filtrate was collected on ice and directly applied to semi-preparative HPLC. The Chl *a* fraction was collected on ice, and the solvents were evaporated *in vacuo*, yielding 9.7 mg of Chl *a*.

HR ESI-MS (positive ion mode): *m*/*z* (found) = 893.5426; *m*/*z* (calculated) = 893.5426, *Δ* = 0.56 ppm.

### TA spectroscopy

Chl *a* samples were prepared by dissolving the pigment in acetone, EtOH or BN to an OD of 0.2–0.3 and then degassing the solution with nitrogen for about one minute.

TA experiments were performed for Chl *a* in acetone, ethanol (EtOH), and benzonitrile (BN). Chl *a* was excited in the B and Q bands in separate experiments for all solvents. The pulse duration of the two pump pulses was comparable, with the B-pump at 20 fs and the Q-pump at 15 fs as measured by SD-FROG and SHG-FROG, respectively. The measured and retrieved FROG traces, as well as the retrieved spectral and temporal phase profiles, are shown in the ESI (Fig. S12†). The TA setups used for B- and Q-band excitation have been described in detail before.^33,34^ Briefly, a 5 kHz, 2.5 mJ, 25 fs amplified laser system (Coherent Legend Elite Duo HE+ Ti : Sapphire MOPA) is used to seed a 1 m long, commercial hollow-core fiber (HCF) with a core diameter of 250 μm filled with 1 bar of Argon gas. The resulting supercontinuum is either frequency-doubled in an achromatic second-harmonic scheme to yield the B-band pump pulse^33^ or spectrally filtered to obtain the Q-band pump pulse.^34^ In the first case, the pump is compressed by a three-prism configuration, while in the second case, the compression is achieved by chirped mirrors. In both experiments, the probe pulse is given by a supercontinuum obtained by seeding a CaF_2_ crystal with the NIR part of the HCF output. For B-band excitation, a double-chopping scheme was employed to reduce scatter, and the pump energy was 60 nJ per pulse with a focal spot diameter of 200 μm; for Q-band excitation, the pump pulse was not chopped and its energy was 16 nJ per pulse with a focal spot of 140 μm. The data was recorded at MA (54.7°) configuration between pump and probe for B- and Q-pumping. Additionally, datasets with parallel and perpendicular polarization of pump and probe were recorded for B-band excitation of Chl *a* in acetone in order to calculate the TAA.

Global analysis was performed with the open-source software Glotaran.^35,36^

### Quantum dynamics simulations

The quantum dynamics simulations were performed on 2D PES at the XMS-CASPT2 (ref. 37–39) level of theory, using the basis set ANO-RCC-VDZP^40–42^ and a CAS(6/6) active space in the gas phase. As coordinates, we chose the normal modes exhibiting the highest overlap with the non-adiabatic coupling vector at the Q_*y*_ minimum geometry,^5^ optimized at the CAM-B3LYP/6-311G* level.^43^ These modes are 171 and 198, lying at 1489 cm^−1^ and 1639 cm^−1^, respectively. Other normal modes (239 and 103) with smaller overlap with the NAC vector and in a different spectral range were also tested for PES construction. Geometries are available in the ESI.† The resulting quantum dynamics is similar for all modes, but the timescales slow down with decreasing overlap with the NAC vector (Fig. S9†). At each point of the PES, a RASSCF calculation was performed with state averaging over four states, followed by one with state averaging over eight states (Fig. S8†). The ensuing XMS-CASPT2 calculation was performed for the states of interest, namely the S_0_, Q_*y*_, Q_*x*_, B_*x*,_ and B_*y*_. An IPEA^44^ and an imaginary shift^45^ of 0.1 were applied, as in our previous study.^5^ With this approach, an energy difference between Q_*x*_ and Q_*y*_ at the FC point of 0.225 eV is obtained, which corresponds well with our reference value of 0.23 eV.^5^ The non-adiabatic coupling elements were computed at the XMS-CASPT2 level of theory. All PES, NAC, and TDM calculations were performed with the OpenMolcas 23.06 program package.^46–48^ The QDng software package^49^ was used for the subsequent quantum dynamic simulations. The propagation was initially performed by assuming a delta excitation into B_*y*_, therefore populating this state's manifold of vibrational levels. Later, an explicitly simulated pump pulse with a maximum field strength of 0.0026 GV cm^−1^, an FWHM of 20 fs, and a central frequency of *ω*_0_ = 3.60 eV was also applied to excite the population from the ground state into the B Band. The laser parameters were chosen to excite a similar population (about 5%) into the B-band as in our experimental studies.

### Calculated ESA spectra

Geometries of the Q_*y*_ and Q_*x*_ states of Chl *a* with one axially coordinated acetone molecule were optimized using Gaussian 16C.02 (ref. 50) with the CAM-B3LYP density functional^51^ and the 6-311G* basis set.^43,52,53^ Bulk solvent effects were described with the polarizable continuum model (PCM).^54,55^ Five roots were included in the optimization. Optimized geometries were verified as energy minima by the absence of imaginary vibrational frequencies.

Excited state absorption spectra for the optimized geometries were simulated at the double-hybrid TDDFT level using the ORCA 5.0.3 software package.^56–58^ The SCS-ωPBEPP86 density functional^59^ was used in the Tamm–Dancoff approximation^60^ along with the def2-TZVPD^61,62^ basis. The calculation was accelerated by employing the RIJCOSX approximation^63,64^ with the def2/J^65^ and def2-TZVPD/C^66^ auxiliary basis sets. A tighter-than-usual integration grid (ORCA keyword DefGrid3) and convergence criteria (ORCA keywords VeryTightSCF TightPNO) were employed. The bulk solvent was described implicitly *via* the linear-response conductor-like polarizable continuum model (LR-CPCM),^67,68^ assuming equilibrium solvation in the excited state.

## Data availability

Data for this article, including absorption spectra, pump pulse spectra, transient absorption and transient absorption anisotropy maps are available at https://zenodo.org/records/14033283.

## Author contributions

EK and AK performed the experiments; SM provided the sample; SR, LB, CD, and RdVR performed calculations; EK, AK, ET, SR, LB, RdVR, CDPD, and JH analyzed and interpreted the data; EK and JH prepared the plots; EK, AK, ET and JH wrote the main text. All authors reviewed the manuscript before submission.

## Conflicts of interest

The authors declare no competing interests.

## Supplementary Material

SC-OLF-D4SC06441K-s001
