# Supplementary material for: Reassessing the role and lifetime of Qx in the energy transfer dynamics of chlorophyll a
Source: Chem Sci. 2024 Nov 27;16(4):1684–95. doi: 10.1039/d4sc06441k (PMC11610765; doi:10.1039/d4sc06441k)
Supplement: SC-016-D4SC06441K-s001 [file SC-016-D4SC06441K-s001.pdf]

Supplementary Information (SI) for Chemical Science.
This journal is © The Royal Society of Chemistry 2024

Supporting Information for
Reassessing the role and lifetime of Q_x in the energy transfer dynamics of Chlorophyll *a*

Erika Keil[1], Ajeet Kumar[1], Lena Bäuml[2], Sebastian Reiter[2], Erling Thyrhaug[1], Simone Moser[3], Christopher D. P. Duffy[4], Regina de Vivie-Riedle[2], and Jürgen Hauer[1]*

[1]Technical University of Munich, Germany; TUM School of Natural Sciences, Department of Chemistry, Lichtenbergstrasse 4, 85748 Garching, Germany.
[2]Department of Chemistry, Ludwig-Maximilians-Universität München, Butenandtstr. 11, 81377 Munich, Germany.
[3]Institute of Pharmacy, Department of Pharmacognosy, University of Innsbruck, Austria.
[4]Digital Environment Research Institute, Queen Mary University of London, London E1 4NS, U.K.
**Jürgen Hauer*

Email: juergen.hauer@tum.de

This PDF file includes:

Supporting text
Figures S1 to S12
Tables T1 and T2
Reference list

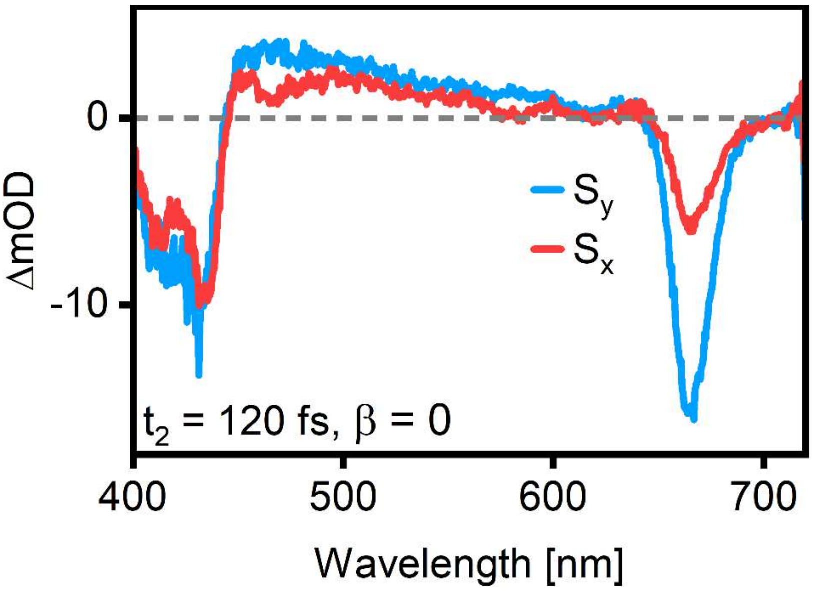

Figure S1: Example for the decomposition of polarized Chl *a* (TA) spectra into x-polarized (S_x) and y-polarized (S_y) components assuming orthogonal TDMs (β = 0˚). The x-polarized spectrum is clearly polluted by y-polarized features: The Q_y band peak is visible in both spectra but should ideally be suppressed in S_x.

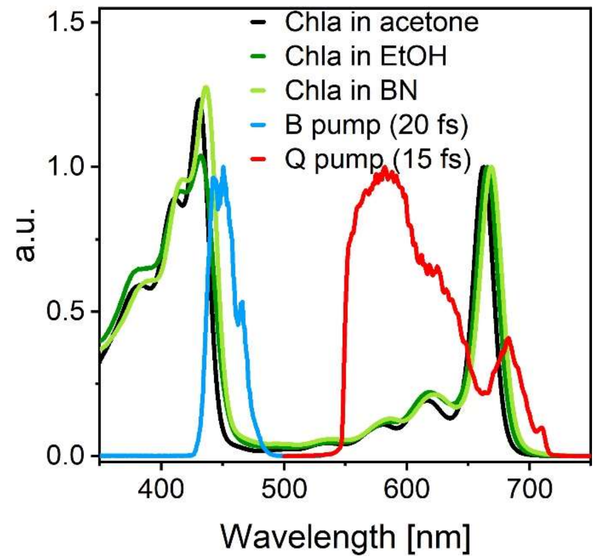

Figure S2: Normalized absorption spectra of Chl *a* in acetone, ethanol (EtOH), and benzonitrile (BN). Separate TA experiments were performed with a 20 fs pump pulse in the blue spectral region (B pump) and a 15 fs pump pulse in the red spectral region (Q pump).

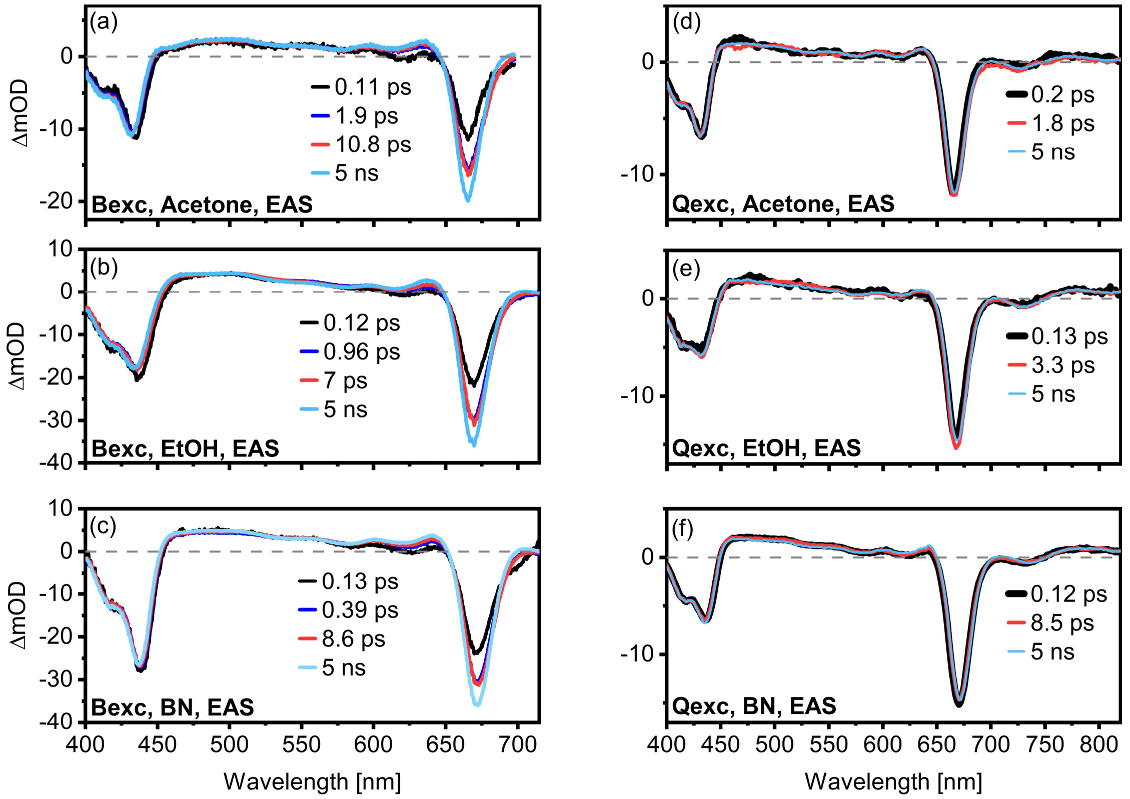

Figure S3: EAS and lifetimes extracted from global analysis of Chl *a* in acetone, EtOH and BN after excitation in the B-band (a-c) and after excitation in the Q-band (d-f).

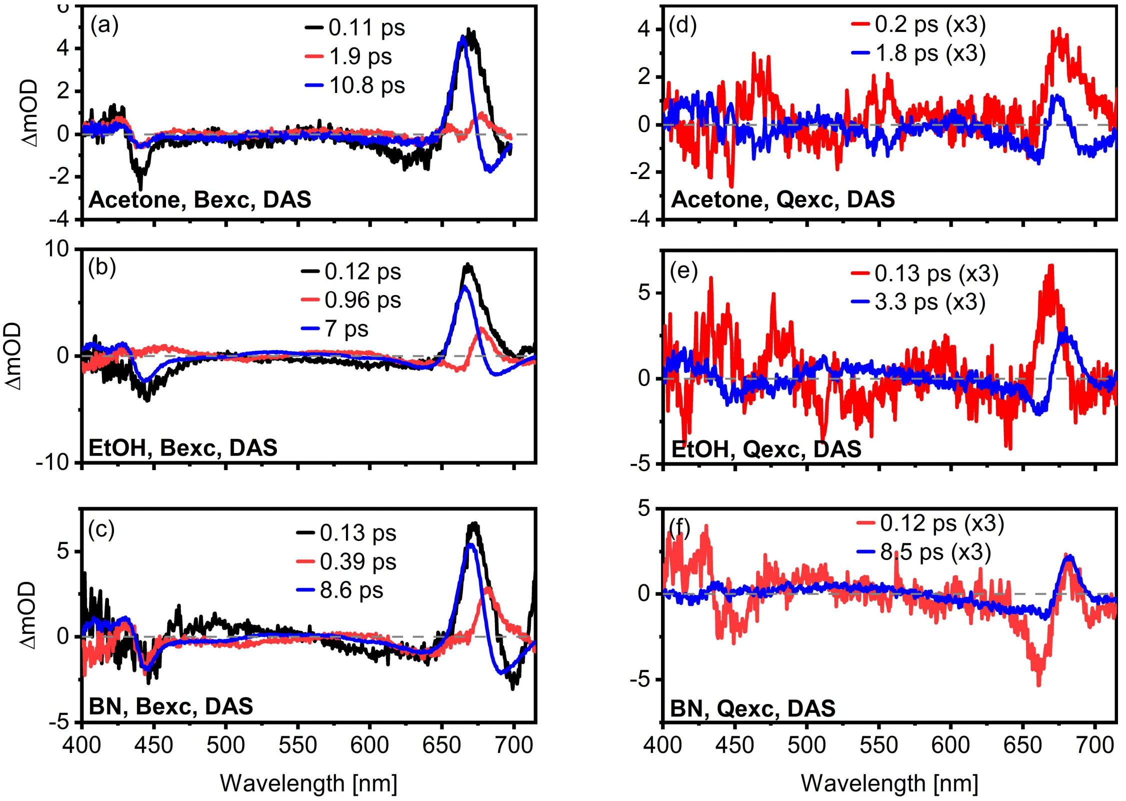

Figure S4: DAS and lifetimes extracted from global analysis of Chl *a* in acetone, EtOH, and BN after excitation in the B-band (a-c) and the Q-band (d-f).

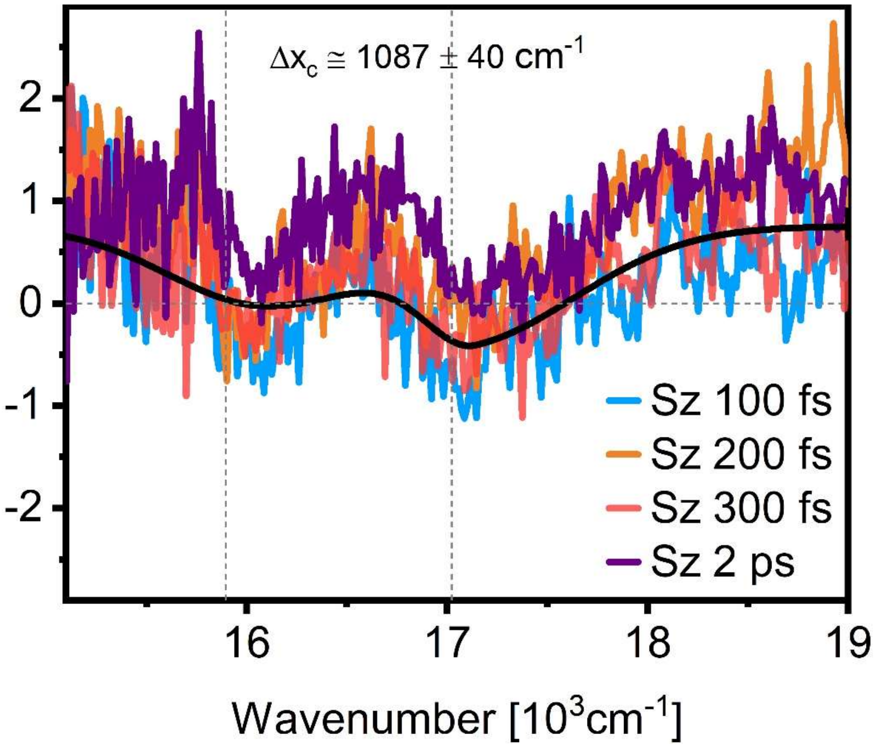

Figure S5: S_x spectra obtained from polarized TA at different time delays. The lineshape remains identical regardless of the population time (bigaussian fit shown in black). The Q_x GSB features are evident, but no Q_x SE or ESA feature is visible at any delay.

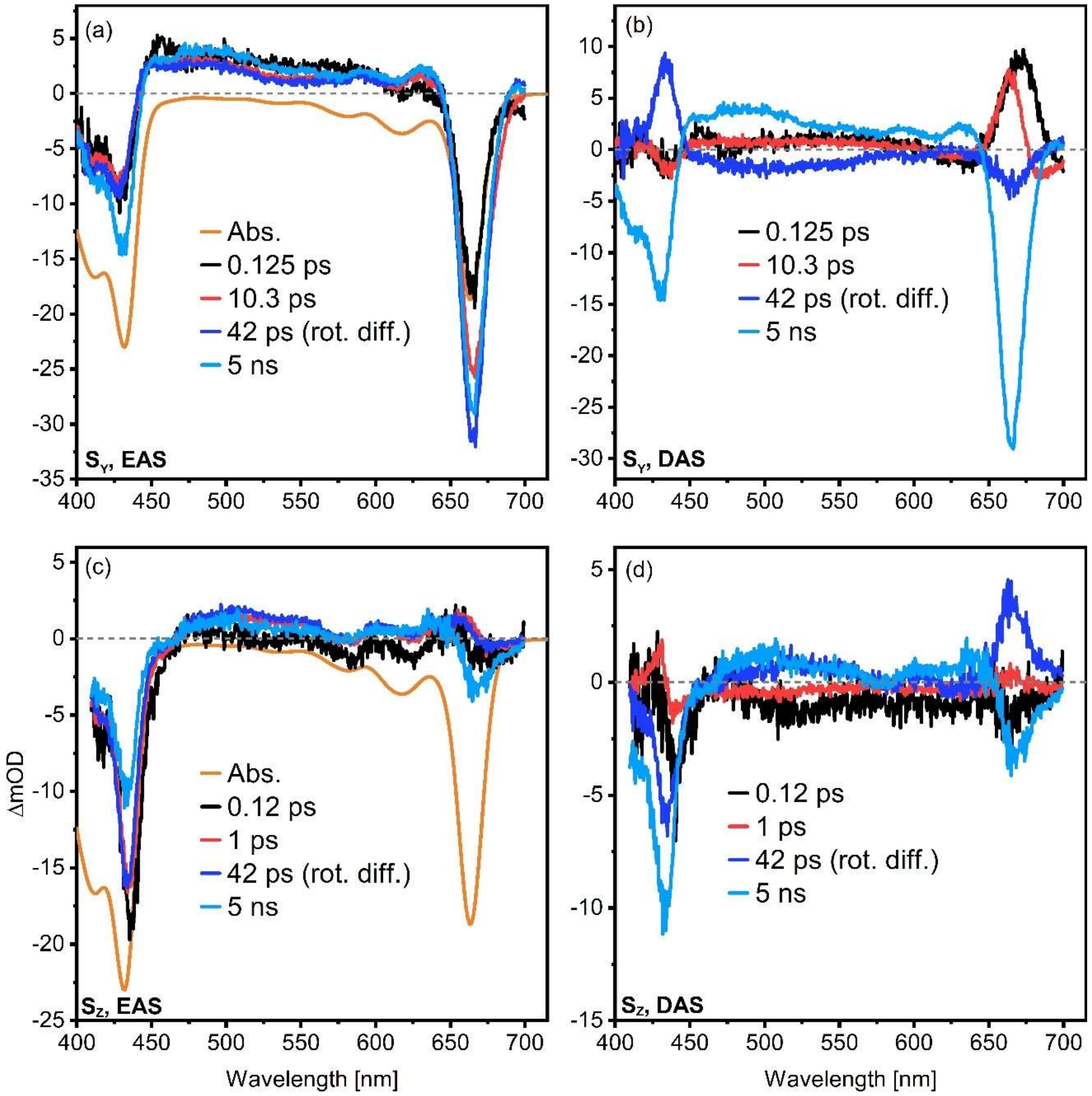

Figure S6: EAS (a, c) and DAS (b, d) from the global analysis of S_Y (top row) and S_X (bottom row) as obtained by decomposition of the polarized TA data of Chl *a*.

Four similar time constants are needed to fit the two datasets. The longest component (5 ns) is just the radiative lifetime of Chl *a*. The second longest component, about 40 ps, is likely the rotational diffusion constant of Chl a in acetone, which can be calculated as 55 ps (1). This is evident in the S_X dataset, in which the Q_Y band is suppressed at early times but comes back with the 40 ps constant. We see rotational diffusion because these are not MA datasets. In the GA for the MA dataset (cf. main text, Figure 2), this component is not there, as expected. The 1-10 ps component corresponds to the 11 ps component in the MA global analysis, which we assigned to some small structural relaxation process. Finally, the shortest process has a lifetime of ca. 120 fs in both datasets and correlates with a loss in the B_X-band GSB region (c) and an increase of the Q_Y amplitude (a). We can, therefore, assign it to B(x)→Q_Y transfer. There is no evidence of an intermediate process including the Q_X band and no spectral features that could be assigned to a Q_X SE or ESA.

Active Space in XMS-CASPT2 Calculations

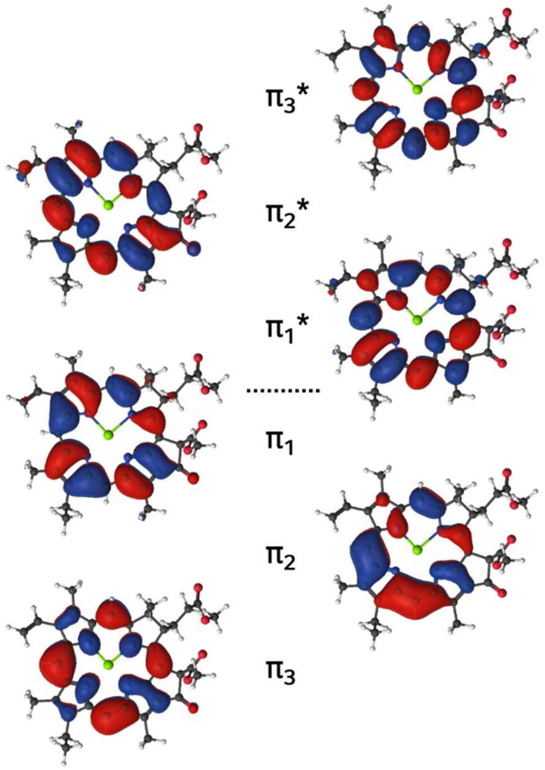

Figure S7: Active space used in the XMS-CASPT2 calculations (Isovalue: 0.02). The active space contains the four Gouterman orbitals (π_2, π_1, π_1*, π_2*) as well as one additional pair of π/π* orbitals.

Coordinate Selection for Quantum Dynamics

The overlap s_i of each normal mode $\mathbf{q_i}$ at the Q_y minimum energy geometry with the non-adiabatic coupling vector $\boldsymbol{f}$ was calculated in Cartesian coordinates according to

$$s_i = \frac{\mathbf{q_i} \cdot \mathbf{f}}{\mathbf{f} \cdot \mathbf{f}}. \tag{1}$$

As the normal modes are orthogonal, the squared overlap s_i^2 provides the weight with which each normal mode contributes to the coupling. The results are summarized in table S10. Cartesian Coordinates of optimized geometries, normal modes and the NAC vector are provided in a zip-archive alongside this document.

Table T1: Overlap of normal modes with the non-adiabatic coupling vector at the Q_y minimum geometry. The harmonic vibrational frequency ν of each mode is also listed.

mode	v [cm^{-1}]	overlap s_i	squared overlap s_i^2
171	1489.52	0.468476	0.219470
198	1639.56	0.336930	0.113522
193	1543.71	0.268399	0.072038
176	1501.62	0.228649	0.052280
197	1633.79	0.225903	0.051032
170	1482.89	0.222164	0.049357
183	1516.32	0.194171	0.037702
154	1358.83	0.166046	0.027571
159	1409.96	0.164634	0.027104
158	1399.21	0.160762	0.025845
184	1517.42	0.151761	0.023032
196	1606.08	0.148694	0.022110
168	1450.34	0.147902	0.021875
157	1393.61	0.139835	0.019554
172	1490.46	0.125946	0.015862
166	1441.60	0.121086	0.014662
160	1419.62	0.117629	0.013836
195	1571.62	0.117460	0.013797
169	1472.45	0.112610	0.012681
192	1533.87	0.105950	0.011225
138	1228.72	0.098410	0.009684
131	1182.08	0.095332	0.009088
179	1511.36	0.091050	0.008290
127	1149.79	0.087146	0.007594
239	3232.21	0.078859	0.006219
136	1216.17	0.078008	0.006085
155	1367.18	0.077860	0.006062
103	940.19	0.076822	0.005902
92	813.35	0.071683	0.005138
109	1014.70	0.070877	0.005024
137	1216.81	0.070459	0.004964
130	1176.90	0.064210	0.004123
147	1313.78	0.058971	0.003478

186	1519.23	0.058032	0.003368
77	706.93	0.055054	0.003031
86	767.21	0.053081	0.002818
141	1246.94	0.052497	0.002756
238	3212.46	0.050883	0.002589
65	516.76	0.050275	0.002528
182	1514.10	0.050030	0.002503
62	485.29	0.049721	0.002472
135	1203.19	0.047865	0.002291
144	1284.54	0.047575	0.002263
102	931.18	0.046559	0.002168
153	1355.47	0.046185	0.002133
185	1518.12	0.045232	0.002046
200	1726.35	0.043242	0.001870
91	800.64	0.042964	0.001846
163	1433.88	0.041305	0.001706
142	1274.88	0.041273	0.001703
145	1300.60	0.040614	0.001649
167	1443.52	0.037995	0.001444
114	1054.02	0.036700	0.001347
187	1519.81	0.036451	0.001329
106	960.34	0.035057	0.001229
132	1195.84	0.034141	0.001166
66	549.12	0.033890	0.001149
156	1375.25	0.032961	0.001086
111	1028.74	0.031913	0.001018
100	890.86	0.031648	0.001002
201	1740.71	0.030857	0.000952
104	948.91	0.030791	0.000948
123	1099.03	0.030051	0.000903
57	412.36	0.029338	0.000861
174	1497.37	0.027636	0.000764
203	1826.63	0.027591	0.000761
151	1339.18	0.025395	0.000645
162	1429.58	0.025260	0.000638
98	883.09	0.024884	0.000619
68	585.57	0.024712	0.000611
60	446.36	0.023613	0.000558
67	575.03	0.023264	0.000541
76	688.90	0.022891	0.000524
108	1001.97	0.022860	0.000523
99	889.88	0.022682	0.000514
84	747.94	0.022526	0.000507
107	977.02	0.022412	0.000502
79	726.43	0.022146	0.000490
56	391.61	0.022092	0.000488
121	1086.82	0.021752	0.000473
70	602.42	0.020914	0.000437
126	1140.56	0.019331	0.000374
119	1076.40	0.018372	0.000338

202	1818.16	0.017645	0.000311
150	1332.28	0.016846	0.000284
52	361.11	0.016638	0.000277
240	3248.77	0.016102	0.000259
83	743.88	0.015620	0.000244
189	1522.15	0.015497	0.000240
199	1654.97	0.014702	0.000216
125	1125.48	0.014557	0.000212
122	1089.25	0.014452	0.000209
194	1556.69	0.014387	0.000207
112	1034.58	0.014193	0.000201
73	652.74	0.013846	0.000192
61	469.02	0.013618	0.000185
128	1152.25	0.013355	0.000178
55	385.00	0.012198	0.000149
113	1044.03	0.012054	0.000145
173	1495.15	0.011597	0.000134
90	799.58	0.011249	0.000127
177	1510.35	0.011083	0.000123
161	1421.81	0.010835	0.000117
80	732.18	0.010788	0.000116
47	302.30	0.010533	0.000111
175	1498.79	0.010456	0.000109
143	1276.56	0.010157	0.000103
190	1523.82	0.010121	0.000102
188	1521.07	0.009975	0.000100
148	1319.48	0.009393	0.000088
72	637.36	0.009208	0.000085
181	1513.06	0.009177	0.000084
205	3049.02	0.008800	0.000077
50	323.22	0.008697	0.000076
25	147.34	0.008680	0.000075
207	3054.00	0.008621	0.000074
58	431.57	0.008437	0.000071
45	292.40	0.008101	0.000066
211	3064.17	0.008043	0.000065
93	818.97	0.008026	0.000064
21	128.13	0.007844	0.000062
149	1326.96	0.007808	0.000061
42	264.18	0.007730	0.000060
37	227.53	0.007649	0.000059
64	506.07	0.007574	0.000057
41	256.67	0.007552	0.000057
97	878.02	0.006917	0.000048
87	772.69	0.006870	0.000047
115	1067.71	0.006863	0.000047
18	112.77	0.006758	0.000046
75	673.66	0.006689	0.000045
88	777.37	0.006579	0.000043
69	588.77	0.006484	0.000042

26	153.06	0.006382	0.000041
146	1300.74	0.006263	0.000039
85	756.34	0.006162	0.000038
63	498.54	0.006037	0.000036
129	1169.97	0.005920	0.000035
27	156.50	0.005857	0.000034
225	3129.78	0.005814	0.000034
43	276.92	0.005756	0.000033
212	3074.01	0.005608	0.000031
116	1069.17	0.005597	0.000031
82	742.24	0.005539	0.000031
206	3052.73	0.005507	0.000030
39	240.13	0.005398	0.000029
54	379.39	0.005319	0.000028
74	664.19	0.005300	0.000028
165	1438.20	0.005192	0.000027
237	3198.74	0.005062	0.000026
204	1849.89	0.004908	0.000024
81	736.21	0.004895	0.000024
46	296.56	0.004845	0.000023
71	632.93	0.004800	0.000023
101	892.81	0.004788	0.000023
140	1231.73	0.004762	0.000023
34	208.25	0.004712	0.000022
234	3166.85	0.004615	0.000021
32	194.86	0.004545	0.000021
78	719.03	0.004413	0.000019
89	787.27	0.004375	0.000019
20	121.56	0.004159	0.000017
14	80.02	0.004023	0.000016
5	28.61	0.003795	0.000014
120	1082.39	0.003780	0.000014
35	212.29	0.003658	0.000013
44	286.85	0.003559	0.000013
110	1024.89	0.003366	0.000011
13	79.83	0.003268	0.000011
208	3055.34	0.003146	0.000010
228	3143.21	0.002989	0.000009
23	136.53	0.002925	0.000009
49	318.99	0.002921	0.000009
22	134.55	0.002791	0.000008
40	251.39	0.002752	0.000008
51	354.82	0.002739	0.000008
218	3098.43	0.002520	0.000006
210	3058.74	0.002482	0.000006
53	374.23	0.002384	0.000006
219	3098.55	0.002312	0.000005
48	308.26	0.002305	0.000005
230	3151.43	0.002279	0.000005
231	3153.20	0.002199	0.000005

164	1435.55	0.002111	0.000004
94	835.85	0.002067	0.000004
10	59.44	0.002051	0.000004
133	1197.06	0.001965	0.000004
24	144.09	0.001921	0.000004
29	176.49	0.001851	0.000003
1	13.23	0.001784	0.000003
139	1230.97	0.001778	0.000003
31	193.04	0.001712	0.000003
38	236.89	0.001691	0.000003
105	957.06	0.001677	0.000003
215	3085.96	0.001584	0.000003
59	434.58	0.001570	0.000002
95	858.33	0.001443	0.000002
96	871.61	0.001436	0.000002
180	1512.75	0.001423	0.000002
19	116.04	0.001419	0.000002
118	1070.66	0.001409	0.000002
36	218.85	0.001376	0.000002
222	3123.63	0.001313	0.000002
220	3100.93	0.001307	0.000002
229	3147.21	0.001230	0.000002
178	1510.99	0.001208	0.000001
2	17.62	0.001206	0.000001
3	21.07	0.001203	0.000001
11	64.79	0.001126	0.000001
217	3094.64	0.001085	0.000001
226	3131.53	0.001075	0.000001
117	1069.33	0.001053	0.000001
6	39.84	0.001040	0.000001
134	1200.53	0.000951	0.000001
33	203.55	0.000856	0.000001
191	1525.15	0.000731	0.000001
227	3140.81	0.000729	0.000001
7	46.69	0.000669	0.000000
9	54.35	0.000655	0.000000
4	24.34	0.000620	0.000000
28	172.27	0.000610	0.000000
30	180.11	0.000561	0.000000
221	3106.76	0.000546	0.000000
12	69.96	0.000509	0.000000
152	1348.32	0.000492	0.000000
8	50.08	0.000434	0.000000
16	98.97	0.000411	0.000000
214	3084.01	0.000368	0.000000
223	3125.75	0.000315	0.000000
15	85.38	0.000313	0.000000
213	3075.25	0.000252	0.000000
236	3194.62	0.000183	0.000000
224	3129.26	0.000170	0.000000

209	3057.03	0.000157	0.000000
17	106.55	0.000128	0.000000
235	3193.42	0.000121	0.000000
124	1106.41	0.000107	0.000000
233	3162.19	0.000050	0.000000
216	3093.82	0.000021	0.000000
232	3158.30	0.000011	0.000000

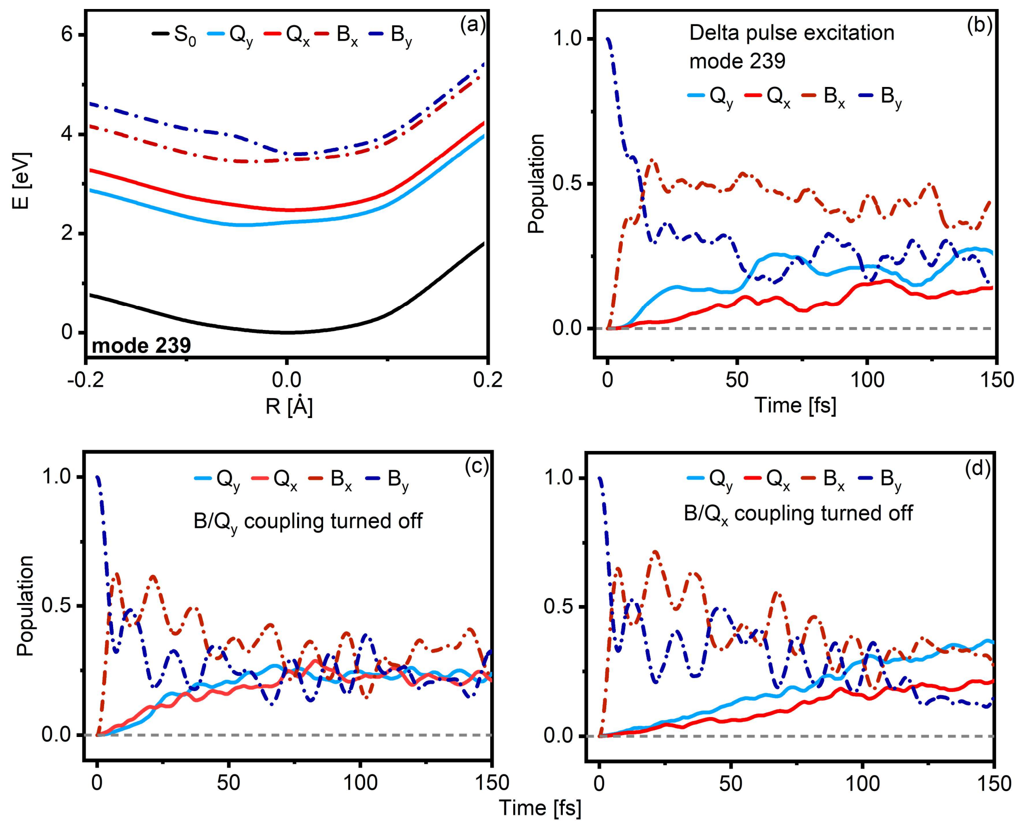

Figure S8: Cuts through the potential energy surfaces of Chl *a* along a mode with a weaker projection on the non-adiabatic coupling vector (a). The relative position of the PES, as well as the population dynamics for the individual states (b), are similar to that obtained along the mode with the strongest NAC projection. The B-to-Q transfer heavily depends on coupling the B band with Q_x. If this coupling is turned off, the B-to-Q transfer slows down significantly (d). On the contrary, if B/Q_y coupling is turned off, the population dynamics do not change much (c). Depicted dynamics are after delta pulse excitation into B_y.

Modeling the effect of VC and IVR

Excitation of Chl *a* with a visible laser pulse means that a lot of energy is deposited into the vibrational modes of the molecule. Energy deposited (directly or indirectly) into a high-lying vibronic state of Qy (v') will relax in a non-linear sequence of intra-molecular vibrational redistribution (IVR), solute-to-solvent energy transfer, and equilibration of the solvent. Explicitly modeling all of these interacting processes is a significant challenge, so we adopt a simplified approach that uses the concept of an effective molecular temperature, T_m. Assuming that IVR is much faster than energy transfer to the solvent, Chl *a* relaxes first from v' to a pseudo-equilibrium to which the elevated molecular temperature T_m can be associated. The assumption of fast thermalization kinetics is based on the fact that the local vibrational density of states is likely to be significant for a large molecule like Chl a. Moreover, we expect the anharmonic couplings between modes to be stronger in the excited state than in the ground state. However, this does not guarantee that thermalization will happen quickly or even at all. The topology of the vibronic manifold can be rather complicated, and the localization of the population on non-thermal trap states can frustrate thermalization (2). In our previous work on carotenoids, the thermalization rate on S_1 occurred on a timescale k_t^{-1} << 1 ps (3), and thermalization times of $k_t^{-1} \sim 1$ ps have been reported for alkanes (2). We consider both cases but don't consider slower thermalization (k_t^{-1} >> 1 ps), as this would violate the implicit time-ordering assumed in our model.

After the thermalization process, vibrational energy is transferred to the solvent. In the steady-state approximation, this can be viewed as a cooling of the molecule until it is at equilibrium with the bulk solvent. We use the approach of Kovalenko et al. (4), where the molecular temperature, T_m, and the temperature of the first solvation shell, T_S evolve according to

$$\frac{dT_m}{dt} = -\gamma N_S \frac{c_S}{c_m}(T_m - T_S) + \left(\frac{dT_m}{dt}\right)_{heat}$$

and

$$\frac{dT_S}{dt} = -\frac{3\chi_S}{{R_S}^2}(T_S - T_{bulk}) + \gamma N_S \frac{c_S}{c_m}(T_m - T_S)$$

The first term on the right-hand side in the first equation represents the collisional energy transfer from the molecule to the solvation shell. N_S is the average number of solvent molecules in the shell, estimated from molecular dynamics (MD) simulations. c_m and c_S are the heat capacities of the molecule and the solvation shell, respectively. The γ parameter is related to the solute-solvent collision rate and is difficult to estimate. Here we obtain a crude estimate using Wheeler's theory of liquids (5), in which the solute is surrounded by solvent molecules that execute spherical positional fluctuations about a fixed center. Overall, we predict a molecule-to-shell transfer time of

$$\tau_{mS} = \frac{1}{\gamma N_S}\frac{c_S}{c_m} \sim 0.4 - 1\text{ps}$$

which is similar to our previous work on carotenoids(3) and to measurements of molecular cooling of stilbene (4). We can additionally define the shell cooling time

$$\tau_{SC} = \frac{R_S^2}{3\chi_S}$$

where R_S is the effective radius of the shell and χ_S is the thermal diffusivity of the solvent. τ_{SC} is 3-5 ps for the solvents considered in this work. The exact values of τ_{mS}, τ_{SC}, and all the parameters used in their calculation are listed in Table S9.

We calculate the evolution of T_m and T_S, assuming thermalization on the timescale of $\sim \tau_{mS}$ ($\sim$ 1 ps) in ethanol, benzonitrile, and acetone. We then fit the evolution of T_m to a bi-exponential function where the two fitted lifetimes, τ_r and τ_{VC}, denote the overall rise (heating) and relaxation (vibrational cooling) times. For all solvents, $\tau_r \sim 1$ ps, and $\tau_{VC} \sim$ 7-9 ps, though with slight variations. The rise and relaxation kinetics for B-band excitation ($E_{Qy,v'} \sim 22,000$ cm^{-1}) and direct excitation of Q_y ($E_{Qy,v'} \sim 20,000$ cm^{-1}) are identical, but in the latter case the maximum T_m is lower. Experimentally, these heating and cooling processes should affect the lineshape of the Chl *a* TA spectra, and we expect to see some thermal broadening of Q_y over $\sim$ 1 ps as the maximum T_m is achieved. The fastest rise in T_m is predicted for benzonitrile, followed by acetone and then EtOH. We then expect the thermal broadening to decrease over $\sim$ 10 ps as the population of low-frequency vibrational modes decreases while the solute and solvent shell re-equilibrate with the solvent.

Again, we predict a solvent dependence for this as molecular cooling and shell cooling (which has a knock-on effect on molecular cooling) depend on solvent properties. We expect this cooling to be slowest for EtOH and fastest for BN, with acetone in the middle. Another important prediction of our simple model is that the relaxation kinetics should be almost independent of whether one excites Q_y directly or indirectly via the B-band. This is because thermalization, even if reasonably slow, is assumed to be much faster than molecular cooling, with the latter having no memory of the non-equilibrium vibrational state before thermalization. However, the maximum T_m reached during the heating-cooling cycle should be affected by excitation wavelength, as this defines the amount of energy thermalized over the low-frequency vibrations. If this were the case, one would predict that, upon direct Q_y excitation, the time constants of energy relaxation would be the same as for B-band excitation, but the overall changes in the lineshape would be much less pronounced.

We stress that the results we discuss in this framework are purely qualitative, as modeling the precise effect of the heating-cooling cycle on the Q_y surface is a significant challenge. For example, we neglect entirely the possibility of direct energy transfer from the non-equilibrium excited state and the solvent. We also ignore the role of extremely low-frequency modes, such as wagging of the phytyl tail or libration of the tetrapyrrole head in defining the pseudo-equilibrium. The mechanisms of molecular and shell cooling are also treated only effectively. Nevertheless, our results point to a promising avenue for further work.

Table T2: Parameters used for the estimation of heat transfer from molecule to shell (τ_{mS}) and of shell cooling (τ_{SC}).

**Parameter**	**Ethanol**	**Benzonitrile**	**Acetone**
N_s	40.7	17.3	27.7
c_S (J K^{-1} mol^{-1})	112	161	126
c_m (J K^{-1} mol^{-1})	529	529	529
γ (ps^{-1})	0.18	0.1	0.15
R_S (nm)	1.03	0.94	0.99
χ_S (nm^2 ps^{-1})	8.71x10^{-2}	9.47x10^{-2}	9.14x10^{-2}
τ_{mS} (ps)	0.37	0.82	1.03
τ_{SC} (ps)	4.03	3.10	3.58

Parameter Generation for the Vibrational Cooling Model

Parameters for the VC and IC model presented in the previous section were generated via MD simulations. For this purpose, a chlorophyll *a* molecule was placed in a cubic simulation box of 10.0 nm edge length and solvated with either acetone, benzonitrile or ethanol. Force field parameters for chlorophyll *a* were taken from the literature (6, 7). Parameters for ethanol, benzonitrile and acetone were generated with antechamber 22.0 (8) using the GAFF2 (9) force field. Partial charges were calculated at the HF/6-31G* level of theory with Gaussian 16 (10) and fitted via restrained electrostatic potential fitting (RESP) (11).

MD simulations were conducted with Gromacs 2023.2 (12). The same protocol was used for each solvent simulation, as described below. First, the energy of the solvated system was minimized with the steepest-descent algorithm until the maximum force fell below 500 kJ mol^{-1} nm^{-1}. The leap-frog integrator was used in all following calculations with a time-step of 2 fs. Bonds to hydrogen atoms were constrained using the LINCS algorithm (13). Verlet lists (14) were used to calculate the short-range electrostatics, using a cutoff distance of 1.2 nm. Long-range electrostatics were evaluated with the smooth particle-mesh Ewald (SPME) method (15, 16) using fourth-order interpolation and a Fourier grid spacing of 0.16 nm. Equilibration was conducted in three stages: First, the system was annealed for 50 ps from 10 K to 100 K in an NVT ensemble and then propagated for another 50 ps at 100 K. The temperature was controlled with the V-rescale thermostat (17) using a time constant of τ_T = 0.1 ps. In the second step, the system was heated to the target temperature of 300 K over 100 ps in an NPT ensemble and propagated for 300 ps at constant temperature and pressure. The pressure was controlled by the Berendsen barostat (18) using an isotropic reference pressure of 1 bar, a time constant of τ_p = 2.5 ps and isothermal compressibilities of 11.19 × 10^{-5} bar^{-1} (ethanol (19)) 4.8 × 10^{-5} bar^{-1} (benzonitrile (20)) and 12.5 × 10^{-5} bar^{-1} (acetone (21)). After temperature and pressure had stabilized, a third equilibration was performed for 4 ns in an NPT ensemble using the Nosé-Hoover thermostat (22, 23) (τ = 2.5 ps) and the Parrinello-Rahman barostat (24, 25) (τ_p = 10 ps). Equilibration was confirmed by the average temperature and pressure converging to their target values and the total energy and density remaining stable. Production simulations were carried out in the equilibrated NPT ensembles for 20 ns and 2000 snapshots were extracted in regular intervals of 10 ps.

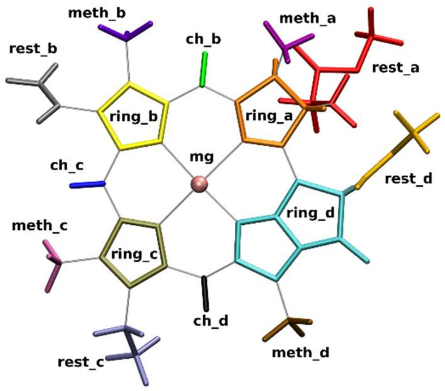

Figure S9: Chlorophyll fragmentation pattern to determine the volume of the first solvation shell. The solvation shell is defined by spheres around the center of each fragment.

To determine the volume of the first solvation shell, the chlorophyll molecule was divided into 16 fragments as depicted in Figure S9. Although it was part of the MD simulations, the phytyl chain attached to the fragment denoted as *rest_a* was not included in the evaluation of the solvation shell. The phytyl chain is mostly unfolded during the MD and rarely comes into contact with the chromophore. The median distance between the outermost carbon atom C20 and the magnesium ion is between 18 Å and 20 Å in all three MD simulations. The distance is greater than 10 Å for over 94 % of the total simulation time. It is therefore reasonable to assume that vibrational cooling into the solvent occurs on a faster or similar timescale than heat dissipation to the phytyl chain. To obtain information about the shape of the first solvation shell (FSS), the radial distribution function (RDF) between each fragment and the surrounding solvent atoms was

calculated and averaged over the entire trajectory. The solvent molecules were modeled by a single reference atom per molecule for the calculation of the RDF (oxygen for ethanol and acetone, nitrogen for benzonitrile). The RDF was normalized and smoothed with a Savitzky–Golay filter (26), using quadratic polynomials and a window size of 15 (ethanol), 20 (acetone) and 25 (benzonitrile) samples. The end of the first peak of the RDF was used to identify the radius of the FSS for each molecular fragment. This allows to construct a sphere around the geometric center of each fragment with the radius of the respective solvation shell. The first frame of each trajectory was used to calculate the fragment centers. The number of solvent molecules in the resulting shape of overlapping spheres (Figure S10) was counted and averaged over the trajectory. A molecule is counted as part of the FSS if its geometric center lies within the solvation sphere of at least one chlorophyll fragment. Moreover, the volume of the solvation shell was estimated numerically and converted to an effective radius of an idealized spherical shell.

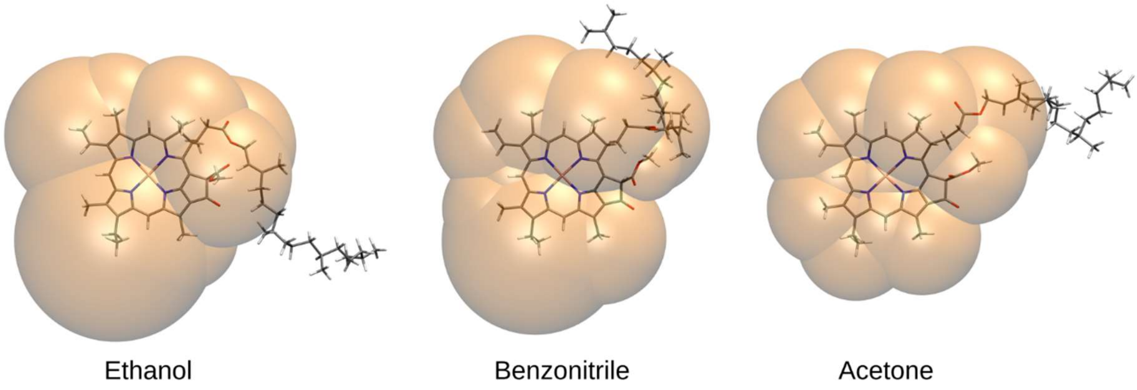

Figure S10: Visualization of the first solvation shell of chlorophyll a in different solvents.

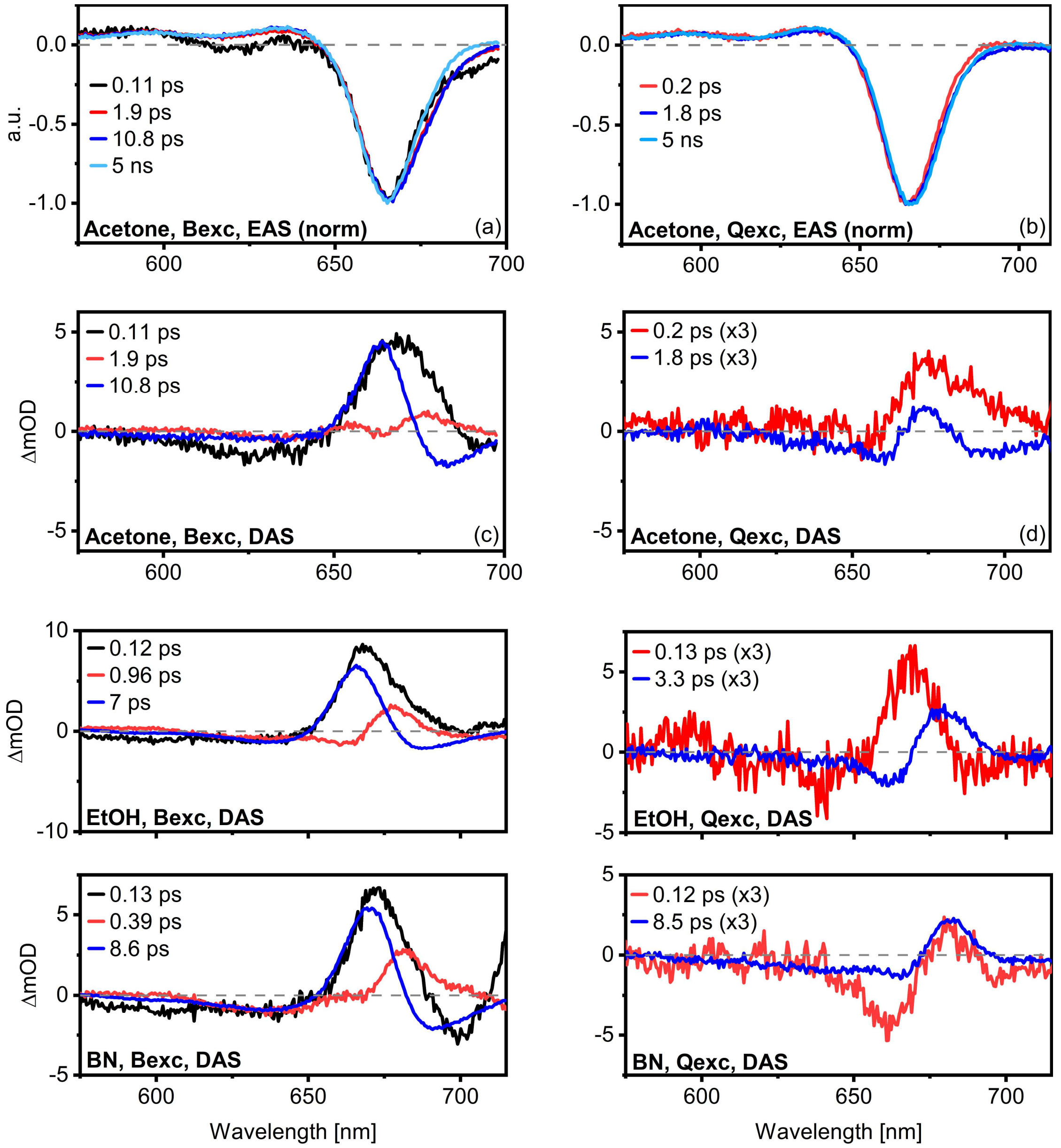

Figure S11: Normalized EAS of Chl *a* in acetone as extracted from GA of Chl *a* after B-band (top left) and Q-band (top right) excitation. A slight broadening is visible on the Q_y red side at early times. The peak then narrows down with the third component (B-band excitation) or broadens and redshifts slightly (Q-band excitation). The lineshape changes are subtle and can be better seen in the DAS. After B-band excitation, the timescales of broadening and narrowing match the expected IVR and VC timescales. After Q-band excitation, the solvent heating and cooling effect, as predicted, is much less pronounced. We see a (slight) continuous broadening and redshift of the peak up to a few ps, a behavior consistent with the Stokes shift dynamics of Chl *a*. The dynamics are qualitatively identical in all three solvents.

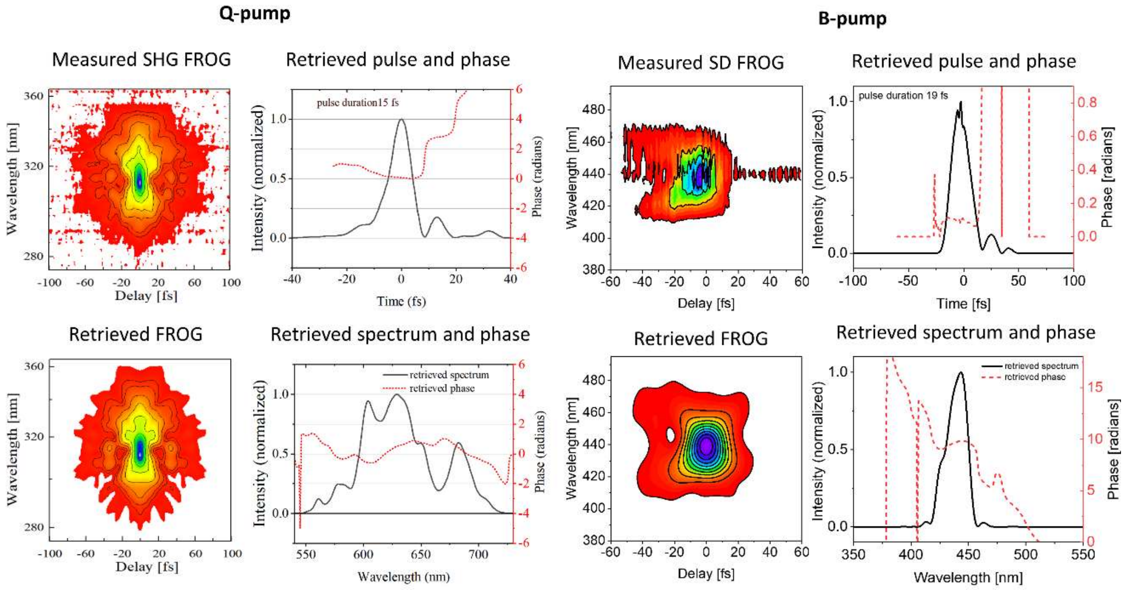

Figure S12: Measured and retrieved FROG traces for the Q- and B-band excitation pulses as measured by SHG-FROG and SD-FROG, respectively. SHG-FROG was measured using a 10 µm thick BBO with a cutting angle of θ = 36.6˚. SD-FROG traces were measured using a 70 µm thick microscope coverglass slide. The retrieved spectra and spectral and temporal phases are plotted separately and yield pulse durations of 15 fs (Q-pump) and 19 fs (B-pump). The pulse duration was obtained with a TG X-FROG retrieval software (FROG 3.1.2, Femtosoft Technologies).

References

1. K. Karki, D. Roccatano, Molecular dynamics simulation study of chlorophyll *a* in different organic solvents. *J. Chem. Theory Comput.* 7, 1131–1140 (2011).
2. D. M. Leitner, Molecules and the Eigenstate Thermalization Hypothesis. *Entropy* 20, 673 (2018).
3. V. Balevičius, *et al.*, The full dynamics of energy relaxation in large organic molecules: From photo-excitation to solvent heating. *Chem. Sci.*10, 4792-4804 (2019).
4. S. A. Kovalenko, R. Schanz, H. Hennig, N. P. Ernsting, Cooling dynamics of an optically excited molecular probe in solution from femtosecond broadband transient absorption spectroscopy. *J. Chem. Phys.* 115, 3256–3273 (2001).
5. T. S. Wheeler, On the theory of liquids. *Proc. Indian Acad. Sci. - Sect. A* 4, 291–297 (1936).
6. M. Ceccarelli, P. Procacci, M. Marchi, An ab initio force field for the cofactors of bacterial photosynthesis. *J. Comput. Chem.* 24, 129–142 (2003).
7. L. Zhang, D. A. Silva, Y. Yan, X. Huang, Force field development for cofactors in the photosystem II. *J. Comput. Chem.* 33, 1969–1980 (2012).
8. J. Wang, W. Wang, P. A. Kollman, D. A. Case, Automatic atom type and bond type perception in molecular mechanical calculations. *J. Mol. Graph. Model.* 25, 247–260 (2006).
9. J. Wang, R. M. Wolf, J. W. Caldwell, P. A. Kollman, D. A. Case, Development and Testing of a General Amber Force Field. *J. Comp. Chem.* 25, 9, 1157-1174 (2004).
10. M. J. Frisch, *et al.*, Gaussian 16 Rev. A.03, Wallingford, CT. (2016).
11. C. I. Bayly, P. Cieplak, W. D. Cornell, P. A. Kollman, A Well-Behaved Electrostatic Potential Based Method Using Charge Restraints for Deriving Atomic Charges: The RESP Model. *J. Phys. Chem.* 97, 40, 10269–10280 (1993).
12. M. J. Abraham, *et al.*, Gromacs: High performance molecular simulations through multi-level parallelism from laptops to supercomputers. *SoftwareX* 1–2, 19–25 (2015).
13. B. Hess, H. Bekker, H. J. C. Berendsen, J. G. E. M. Fraaije, LINCS: A Linear Constraint Solver for molecular simulations. *J. Comput. Chem.* 18, 1463–1472 (1997).
14. S. Páll, B. Hess, A flexible algorithm for calculating pair interactions on SIMD architectures. *Computer Physics Communications* 184, 2641–2650 (2013).
15. T. Darden, D. York, L. Pedersen, Particle mesh Ewald: An N·log(N) method for Ewald sums in large systems. *J. Chem. Phys.* 98, 10089–10092 (1993).
16. U. Essmann, *et al.*, A smooth particle mesh Ewald method. *J. Chem. Phys.* 103, 8577–8593 (1995).
17. G. Bussi, D. Donadio, M. Parrinello, Canonical sampling through velocity-rescaling. *J. Chem. Phys.*126, 014101 (2008).
18. H. J. C. Berendsen, J. P. M. Postma, W. F. Van Gunsteren, A. Dinola, J. R. Haak, Molecular dynamics with coupling to an external bath. *J. Chem. Phys.* 81, 3684–3690 (1984).
19. D. R. Lide, *et al.*, CRC Handbook of Chemistry and Physics. CRC Press/Taylor & Francis Group, Boca Raton, FL. (2007).
20. C. Caleman, *et al.*, Force field benchmark of organic liquids: Density, enthalpy of vaporization, heat capacities, surface tension, isothermal compressibility, volumetric expansion coefficient, and dielectric constant. *J. Chem. Theory Comput.* 8, 61–74 (2012).
21. W. A. Adams, K. J. Laidler, Effects of pressure and temperature on the structure of liquid acetone. *Can. J. Chem.* 45, 123–130 (1967).
22. W. G. Hoover, Canonical dynamics: Equilibrium phase-space distributions. *Phys. Rev. A* 31, 1695–1697 (1985).
23. S. Nosé, A molecular dynamics method for simulations in the canonical ensemble. *Mol. Phys.* 52, 255–268 (1984).

24. M. Parrinello, A. Rahman, Polymorphic transitions in single crystals: A new molecular dynamics method. *J. Appl. Phys.* 52, 7182–7190 (1981).
25. S. Nosé, M. L. Klein, Constant pressure molecular dynamics for molecular systems. *Mol. Phys.* 50, 1055–1076 (1983).
26. A. Savitzky, M. J. E, Smoothing and Differentiation of Data by Simplified Least Squares Procedures. *Anal. Chem.* 36, 8, 1627–1639 (1964).
